# The adaptation of *Fusarium culmorum* to DMI Fungicides Is Mediated by Major Transcriptome Modifications in Response to Azole Fungicide, Including the Overexpression of a PDR Transporter (FcABC1)

**DOI:** 10.3389/fmicb.2018.01385

**Published:** 2018-06-26

**Authors:** Pierre Hellin, Robert King, Martin Urban, Kim E. Hammond-Kosack, Anne Legrève

**Affiliations:** ^1^Earth and Life Institute, Applied Microbiology, Phytopathology, Université Catholique de Louvain, Louvain-la-Neuve, Belgium; ^2^Department of Computational and Systems Biology, Rothamsted Research, Harpenden, United Kingdom; ^3^Department of Biointeractions and Crop Protection, Rothamsted Research, Harpenden, United Kingdom

**Keywords:** ABC transporter, triazoles, fungicides, RNA-Seq, crop protection

## Abstract

*Fusarium culmorum* is a fungal pathogen causing economically important diseases on a variety of crops. Fungicides can be applied to control this species with triazoles being the most efficient molecules. *F. culmorum* strains resistant to these molecules have been reported, but the underlying resistance mechanisms remain unknown. In this study, a tebuconazole-adapted *F. culmorum* strain was developed with a level of fitness similar to its parental strain. The adapted strain showed cross-resistance to all demethylation inhibitors (DMIs), but not to other classes of fungicides tested. RNA-Seq analysis revealed high transcriptomic differences between the resistant strain and its parental strain after tebuconazole treatment. Among these changes, *Fc*ABC1 (FCUL_06717), a pleiotropic drug resistance transporter, had a 30-fold higher expression level upon tebuconazole treatment in the adapted strains as compared to the wild-type strain. The implication of this transporter in triazole resistance was subsequently confirmed in field strains harboring distinct levels of sensitivity to triazoles. *FcABC1* is present in other species/genera, including *F. graminearum* in which it is known to be necessary for azole resistance. No difference in *FcABC1* sequences, including the surrounding regions, were found when comparing the resistant strain to the wild-type strain. *Fusarium culmorum* is therefore capable to adapt to triazole pressure by overexpressing a drug resistance transporter when submitted to triazoles and the same mechanism is anticipated to occur in other species.

## Introduction

*Fusarium culmorum* ([W.G. Smith] Saccardo) is a fungal pathogen responsible for multiple diseases on a variety of crops and weeds. Among these diseases, Fusarium head blight (FHB) is one of the major small-grain cereal diseases. It is caused by a complex of species belonging to the *Fusarium* and *Microdochium* genera that can infect the ear (Parry et al., 1995). *Fusarium* spp. are the species of most concern because, in addition to yield losses, they can produce a wide range of mycotoxins (Desjardins, 2006). *Fusarium culmorum* and *F. graminearum* (Schwabe), a closely related species, are among the most common species found in Europe, but with high variations between years and locations (Parry et al., 1995; Xu et al., 2005; Hellin et al., 2016a). These species can produce the type B trichothecenes deoxynivalenol (DON) or nivalenol (NIV), depending on the strain. Type B trichothecenes were shown to contribute to virulence on wheat and were demonstrated to inhibit protein translation threatening human and animal health (Desjardins, 2006).

Triazoles are the most widely used fungicides in managing FHB because of their greater efficacy compared with other active ingredients (Mesterházy, 2003). As demethylation inhibitors (DMIs), they prevent fungal growth by specifically binding to the 14α-demethylase (CYP51), an essential enzyme for completing the synthesis of ergosterol (Ziogas and Malandrakis, 2015). Resistance levels to triazoles of *F. graminearum* populations have been shown to have gradually increased since their introduction into the market (Klix et al., 2007; Sun et al., 2014), and resistant strains of *F. graminearum* have also been reported in China and the US (Yin et al., 2009; Spolti et al., 2014). In addition, prolonged laboratory exposure to triazoles caused the adaptation of *Fusarium* spp. resulting in the appearance of resistant phenotypes (Becher et al., 2010; Serfling and Ordon, 2014). Although high variations in sensitivity have been noted among strains of *F. culmorum*, no decline of sensitivity has been observed over time, probably because of a fitness cost associated with the resistance (Hellin et al., 2016b).

In various fungi, an increase in fungicide resistance has been achieved by: (1) target gene duplication; (2) mutation of the target protein; (3) overexpression of the target gene; (4) fungicide detoxification or; (5) fungicide export by transporters (Lucas et al., 2015; Ziogas and Malandrakis, 2015). The molecular mechanisms involved in resistance to triazoles in *Fusarium* spp. are still unknown and are mostly investigated in *F. graminearum*. The genome of this species has revealed the presence of three paralogous copies of the *CYP51* gene, among which *CYP51A*and *CYP51B*, are thought to be involved in triazole sensitivity (Liu et al., 2011; Fan et al., 2013; Qian et al., 2018). Laboratory directed mutation Y137H in *F. graminearum CYP51B* resulted in reduced sensitivity to tebuconazole (Qian et al., 2018). Nevertheless, neither mutation nor overexpression of those gene, has been related to a change in azole sensitivity in *F. graminearum* (Yin et al., 2009; Talas and McDonald, 2015). Transporters from the ATP-binding cassette (ABC) family are known for their implication in drug resistance in multiple fungi (Ziogas and Malandrakis, 2015) and *Fusarium graminearum* possesses 62 potential ABC transporters (King et al., 2015). The function of four of them has been investigated through gene deletion, and *FgABC3* (FGSG_04850) has been shown to be essential for azole resistance (Abou Ammar et al., 2013). Moreover, a homologous copy of this gene (FcABC1) has been previously found to be necessary for full virulence in *F. culmorum* (Skov et al., 2004).

The use of “omics” analysis for the determination of resistance mechanisms is gaining interest and it is anticipated to play a big role in the future fungicide development and registration procedures (Cools and Hammond-Kosack, 2013). For example, genome-wide transcriptomic analysis were used to establish *F. graminearum* response to azole treatment and revealed the overexpression of multiple genes, including transporters (Liu et al., 2010; Becher et al., 2011). In other fungal species, comparison of the transcriptomic response to azole of strains with different levels of sensitivity allowed the identification of transporters for which the level of expression could be linked to resistance (Hulvey et al., 2012; Omrane et al., 2015; Sang et al., 2015). The recent publication of the first annotated version of the *F. culmorum* strain UK99 (Urban et al., 2016) provides a new tool to investigate triazole resistance mechanisms in this agro-economically important species.

In this study, we adapted the sensitive *F. culmorum* UK99 strain to tebuconazole *in vitro* and compared its transcriptome with that of its parental strain when subjected to this fungicide. By using strains that shared the same genetic background, the differences detected could be directly attributed to DMI adaptation and were verified on a set of field strains harboring distinct levels of triazole sensitivity.

## Materials and methods

### Strains and preservation

*Fusarium culmorum* strain UK99 (syn. NRRL 54111 and FGSC 10436) was used as the reference strain in this study. Six other *F. culmorum* strains, either sensitive (MBC 7964, MBC 6020, and MBC 7603) or more resistant (MBC 190, MBC 7555 and CRA-PAV ER 1998) to triazoles, as previously characterized (Hellin et al., 2016b), were used as field controls. All strains were stored in 20% glycerol at −80°C.

### Adaptation of *Fusarium culmorum* to tebuconazole

*Fusarium culmorum* strain UK99 was adapted to tebuconazole following the protocol described by Becher et al. (2010), with slight modifications. In brief, three plugs (5 mm diameter) from UK99 cultured on potato dextrose agar (PDA; Oxoid, Waltham, MA, USA) were used to inoculate 100 mL of potato dextrose broth (PDB; Becton Dickinson, Franklin Lakes, NJ, USA) cultured in the dark at 22°C (100 rpm). After 3 days, the media were replaced by fresh PDB amended with 0.4 mg/L of tebuconazole (Pestanal; Sigma Aldrich, Saint-Louis, MI, USA) dissolved in ethanol. After 11 days, the media were replaced again with fresh PDB amended with 1 mg/L and 11 days later with PDB containing 10 mg/L of tebuconazole. Mock cultures were supplied at the same time, with fresh PDB supplemented with the same volume of ethanol (1%). The mycelia produced were harvested by centrifugation (3,000 rpm for 10 min) and homogenized in twice their volume of 20% glycerol with a Sorvall Omni-Mixer (DuPont Instruments, Wilmington, DE, USA) at half speed for 10 s. Conidia were produced by inoculating 100 μL of each culture in mung bean broth (MBB, 40 g of mung beans per liter) and cultivated at 22°C with a 12 h alternation of light and darkness for 7 days (50 rpm). These suspensions were serially diluted and spread onto PDA plates supplemented with 10 mg/L of tebuconazole and incubated at 22°C. Colonies that subsequently grew were transferred to new PDA plates. Single conidium strains were then produced and cultured in MBB to produce the first generation of adapted strains. Each strain was cultivated on PDA for 5 days and then a new PDA plate was inoculated with a plug originating from the margin of the colony. This last procedure was repeated six times to produce a seventh generation that was used to test the mitotic stability of the fungicide resistance acquired by the strain.

### Assessment fitness parameters and pathogenicity

A rapid screening procedure of the adapted phenotypes of the first generation was carried out in order to find a suitable candidate strain comparable with the parental strain (UK99). A preliminary test compared growth rate, sporulation and pathogenicity to wheat of the strains, as explained hereafter. Fitness parameters of the wild-type strain (UK99) and the selected tebuconazole-adapted strain (P1P2) of the first generation were then more thoroughly assessed by other assays. The growth rate of each strain was determined by measuring colony radius after 4 days on PDA medium inoculated with a 5 mm plug excised from the margin of a 7-day-old culture. Experiments were performed at 10, 22, and 30°C with three plates per condition and two biological replicate. Sporulation capacity was determined by measuring the conidia concentration obtained in MBB, as explained above, with four replicates and two biological repeats.

In order to assess the pathogenicity of strains toward wheat seedlings (cv Homeros), disinfected seeds (5 min in 1% NaOCl, rinsed twice in sterile water for 2 min and dried on filter paper) were deposited in test tubes filled with 6 mL of 1.5% water agar (1 seed/tube). The tubes were kept in the dark for 3 days to allow germination and then inoculated with a mycelial plug (5 mm). Uncolonized PDA plugs were used for mock controls. The length of the plantlets was measured at 4 and 11 days post-inoculation and growth rate retardation was calculated with regard to control plantlets. Two replications of 10 plantlets per strain were used.

Wheat seeds of the spring cv Triso were sown in 20 cm pots (2 seeds/pot) filled with a mixture containing equal parts of local agricultural soil, sand and potting mix. The plants were grown in a greenhouse and inoculated as described by Fernandez and Chen (2005). Half of the plants were then treated with tebuconazole (Horizon, Bayer) using a hand sprayer (1.25 L Comfort 814; Gardena, Ulm, Germany). The concentration of the active ingredient in the mixture in order to achieve the recommended spraying rate of 1 L/Ha was initially estimated to be 0.25 mg/mL. After 14 days, ear surface presenting bleached symptoms on were evaluated. Fourteen ears were scored per modality.

The pathogenicity to maize seedlings of strains UK99 and the selected strain P1P2 was assessed on the maize hybrid “Troizi CS.” Ten-day-old cultures on Corn Meal Agar (CMA; corn grits [200 g/L] and agar [20 g/L]) were blended and incorporated in 500 mL of a sterile mixture of potting mix and sand (2:1). Two culture plates were used per 20 cm pot. After 3 days of incubation, 10 grains of maize (surface sterilized as previously explained) were sown in each pot and cultivated in the greenhouse (20°C). Seedling length was measured after 15 days and compared with the controls.

The same maize cultivar was used to compare the pathogenicity of both strains toward maize stalks using toothpick inoculation assays, as described by Scauflaire et al. (2012), with nine plants per modality. Necrotic areas observed in longitudinal cross section were measured with ImageJ software v. 1.48 (Schneider et al., 2012) and the mean area of the controls was subtracted from the ones of the inoculated stem sample.

### Quantitative and qualitative determination of fungicide sensitivity

The sensitivity of the first and seventh generations of UK99 and P1P2 to tebuconazole (Horizon, Bayer) was assessed using a microtiter plate assay (Hellin et al., 2016b) to quantify the increase in triazole resistance acquired through the adaptation procedure. In order to test for cross-resistance with other classes of fungicides, about 250 conidia (2 μL of a 1.25 × 10^5^ conidia m/L spore suspension) were point inoculated onto PDA plates amended with fungicide at concentrations preliminary estimated to inhibit UK99 growth by about 80-95%. The fungicides used for comparison were tebuconazole (1 mg/L; Horizon, Bayer), epoxiconazole (1 mg/L; Opus, BASF), cyproconazole (10 mg/L; Caddy, Bayer), Bayer), tetraconazole (10 mg/L; Eminent, Isagro), propiconazole (1 mg/L; Tilt, Syngenta), difenoconazole (1 mg/L; Score, Syngenta), prothioconazole (10 mg/L; Proline, prochloraz (0.1 mg/L; Sportak, BASF), imazalil (1 mg/L; Fungaflor, Certis Europe), fenarimol (10 mg/L; Rubigan 40, Dow), carbendazim (1 mg/L; Bavistin, BASF), thiabendazole (1 mg/L; Tecto, Syngenta), pyrimethanil (100 mg/L; Scala, BASF), and fenpropimorh (100 mg/L; Corbel, BASF). The fungicides were dissolved in ethanol, apart from Opus, Tecto and Bavistin, which were dissolved in water because of their commercial formulation. The plates were incubated in the dark at 22°C for 7 days. Both sensitivity tests contained triplicates and were repeated twice. Azoxystrobin (Amistar, BASF), boscalid (Cantus, BASF) and bixafen (Pestanal; Sigma Aldrich) were omitted from the test because the maximum growth reduction obtained with 100 mg/L of these fungicides on UK99 was only 61, 22, and 65%, respectively.

### RNA isolation and RT-qPCR

For each strains, 10^6^ conidia were used to inoculate 100 mL of liquid complete medium (CM, Correll et al., 1987). The cultures were incubated in the dark (22°C, 120 rpm) for 24 h before being supplemented with 0.5 mL of tebuconazole (Pestanal; Sigma-Aldrich) dissolved in ethanol to reach a concentration of 2.5 mg/L. The same amount of ethanol was added to the control cultures. After 12 h, mycelium was harvested from each culture (three per condition) in Miracloth and quickly washed with sterile water before being flash frozen in liquid nitrogen and ground with a mortar and pestle. RNA extraction was performed on 100 mg of sample using RNeasy Plant mini kit (Qiagen, Hilden, Germany) combined with the RNase-free DNase set (Qiagen). Total RNA was reverse-transcribed with M-MLV Reverse Transcriptase kit (Promega, Madison, WI, USA) using a cocktail of anchored oligo(dT)_25_. Quantitative PCRs were performed using Takyon No Rox SYBR MasterMix dTTP Blue reactive (Eurogenetec, Seraing, Belgium) in CFX96 thermal cycler (BioRad, Hercules, CA, USA). The expression of two genes coding for predicted cofilin and pre-mRNA splicing factor and known for their stability in *F. graminearum* under fungicide stress (FGSG_06245 and FGSG_01244, respectively; Becher et al., 2011) were used as housekeeping genes. Normalization of expression was performed by the 2^−ΔCt^ method implemented in CFX manager software (BioRad). All primer pairs were obtained using primer-BLAST and were designed so that one primer would span an exon junction, when it was possible (Table S5). Standard PCR and gel electrophoresis were performed to verify the presence of a single amplicon after amplification of cDNA samples and the absence of genomic amplification on total RNA extracts. For each primer pair, the amplification product was purified using MSB spin PCRapace (Startec Biomedical AG, Birkenfeld, Germany) and sequenced to confirm target specificity (Macrogen Europe, Amsterdam, The Netherlands).

### Library preparation and sequencing

Samples of total RNA belonging to tebuconazole-treated cultures of UK99 and P1P2 (three replicates per strain) were sent to Beckman Coulter Genomics facility (Danvers, MA, USA). Total RNA quantity and quality were determined with a Quant-iT RNA assay kit (Invitrogen, Carlsbad, CA, USA) and a TapeStation (Agilent Technologies, Santa Clara, CA, USA), respectively. The six libraries were prepared using TruSeq RNA Library Prep Kit v2 (Illumina, San Diego, CA, USA) on a Biomek FX^P^ liquid handling platform (Beckman Coulter, Brea, CA, USA). Qualitative and quantitative evaluation of the content of each library was performed using the KAPA library quantification kit for Illumina platform (Kapa Biosystems, Boston, MA, USA) and a Tapestation, respectively. Libraries were then diluted to 25 pM, pooled and sequenced (2 × 125 pb) on Illumina HiSeq2500 platform (high output mode) using V4 reagents. The quality of sequencing was assessed using FastQC (Andrews, 2016).

### Transcriptome analysis

Hisat2 (Pertea et al., 2016) was used to map the reads to *F. culmorum* UK99 genome (Accession No. PRJEB12835; Urban et al., 2016) with default settings and using the genome annotation (http://pre.fungi.ensembl.org). No pre-processing of the reads took place. Cuffdiff v2.2.1 (Trapnell et al., 2013) was used determine the abundance of transcripts in FPKM (Fragments Per Kilobase per Million mapped reads). A differential testing of counts (P1P2 vs. UK99) was performed with Cuffdiff with a classic FPKM library normalization method, pooled dispersion estimation method, and bias correction with effective length, counting against the UK99 gene annotation only. Annotations were added to the Cuffdiff output using Blast2go v3.3.5 (Conesa et al., 2005) with the Blast2go GO database (05/2016) and default filtering settings, using input from Interpro (v58.0) and DeCypher (Timelogic, USA, CA, Carslbad) with the NCBI NR database (23/06/16) using an e-value threshold of 1e-2. SNP calling was performed with FreeBayes (version 1.0.2.29) using default settings, filtering for minimum coverage of 10 reads. SNP effects were predicted using snpEff (4.0). Heterozygous SNPs were considered as low frequency variant and were not further analyzed. Data were compared to that of a microarray study comparing the transcriptome of *F. graminearum* PH-1 with or without tebuconazole application (Becher et al., 2011; E-GEOD-25114).

### Sequencing of *FcABC1*

DNA of *F. culmorum* strains was extracted from 100 mg of ground mycelia using DNeasy Plant Mini kit (Qiagen). Primers for sequencing of gene *FcABC1* and its flanking regions were designed with primer-Blast (Table S6). Two fragments corresponding to the upstream region (about 1,000 bp from the start codon) and the ORF + partial 3′ UTR region (about 5,000 bp) were amplified using Phusion High-Fidelity DNA Polymerase (Thermo Scientific, Waltham, MA, USA) following the manufacturer's instructions. Amplicons were purified with MSB spin PCRapace kit (Startec Biomedical AG) and sequenced by Macrogen using the amplification primers. Six intermediate primers were used for sequencing the ORF + 3′ UTR region because of its extensive length (Table S6). Only the promoter region was amplified for *F. culmorum* field isolates. Sequences were assembled and contigs were subsequently edited with Sequencher 4.8 (Gene Codes Corp., Ann Arbor, MI, USA). After alignment with MUSCLE, DNA sequence of UK99 was compared manually to the ones of P1P2, *F. culmorum* CS7071 (NCBI Accession No. CBMH000000000; Moolhuijzen et al. 2013)*, F. graminearum* PH-1 (FGSG_04850 = FgABC3) and *F. pseudograminearum* CS3096 (FSPE_06011; Moolhuijzen et al., 2013).

### Statistical analysis of fitness parameters and RT-qPCR

Comparisons of fitness parameters between P1P2 and UK99 were analyzed using *t*-tests or two-way ANOVA (depending on the number of factors) in JMP® PRO 12 (SAS Institute Inc., Cary, NC, USA). EC_50_ values were determined using logistic regression as described by Hellin et al. (2016b) and compared among strains and generations by a two-way ANOVA. Quantitative real-time PCR results between P1P2 and UK99 were compared using two-way ANOVA. Differences in expression of selected genes in sensitive and resistant field strains were analyzed using linear mixed models with the “strain” set as a random factor. Whenever the interaction parameter was significant, Tukey's HSD test was performed.

## Results

### *Fusarium culmorum* is able to adapt to tebuconazole without losing its competitiveness

**Figure 1:**
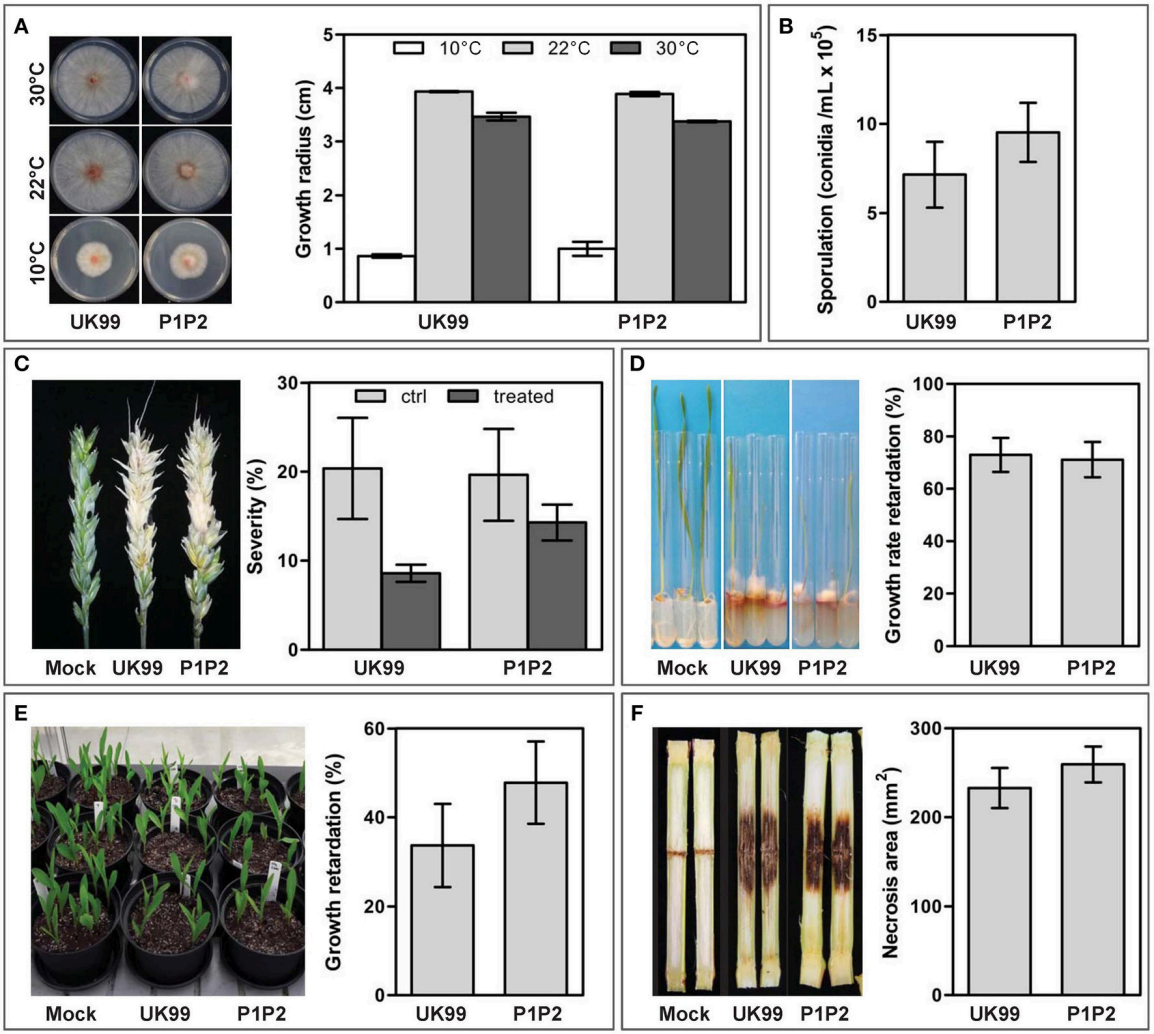
Evaluation of the fitness parameters of the tebuconazole-adapted strain (P1P2) and its parental strain (UK99). Comparison between the strains was based on **(A)** growth rate, **(B)** sporulation capacity, **(C)** pathogenicity on wheat ears with (treated) and without (ctrl) tebuconazole treatment, pathogenicity to **(D)** wheat and **(E)** maize plantlets and **(F)** aggressiveness toward maize stalk. Error bars represent standard errors.

After 35 days, the cultivation of *F. culmorum* strain UK99 in liquid broth, supplemented with periodically-increasing concentrations of tebuconazole, yielded multiple resistant colonies, which subsequently grew on potato dextrose agar amended with tebuconazole (10 mg/L). The strain P1P2 was selected for its characteristics similar to those of the wild-type strain (Figure S1). The resemblance of both strains was further investigated with multiple assays (Figure 1). After 4 days of incubation on PDA, although temperature had a significant impact on strain growth (*P* < 0.001, Figure 1A), the radius of both strains were similar (*P* = 0.533). Sporulation capacity of the adapted strain also remained unchanged (*P* = 0.355, Figure 1B). Both strains were found to be equally aggressive to both wheat and maize. They produced similar discoloration on wheat ears, with symptoms covering an average surface of 20% of the spikes (*P* = 0.535, Figure 1C). A tebuconazole treatment applied 2 days after inoculation resulted in the symptoms being reduced by half (*P* = 0.037), but no significant difference was observed between strains (*P* = 0.535), although P1P2 seemed to be a little more competitive than UK99 on treated ears (Figure 1C). Growth rate of the wheat plantlets, measured between 4 and 11 days after inoculation, was reduced in a similar way by both strains, with an average of 72% reduction (*P* = 0.846, Figure 1D). Maize seedling growth after 15 days was reduced by an average of 40% by both strains (*P* = 0.287; Figure 1E) when the seeds were sown in infected soil. In maize stalk infection assays, the average surface of necrotic lesions produced by both strains was also similar (*P* = 0.391).

### The adapted strain is more resistant to DMI fungicides than the wild-type strain

**Figure 2:**
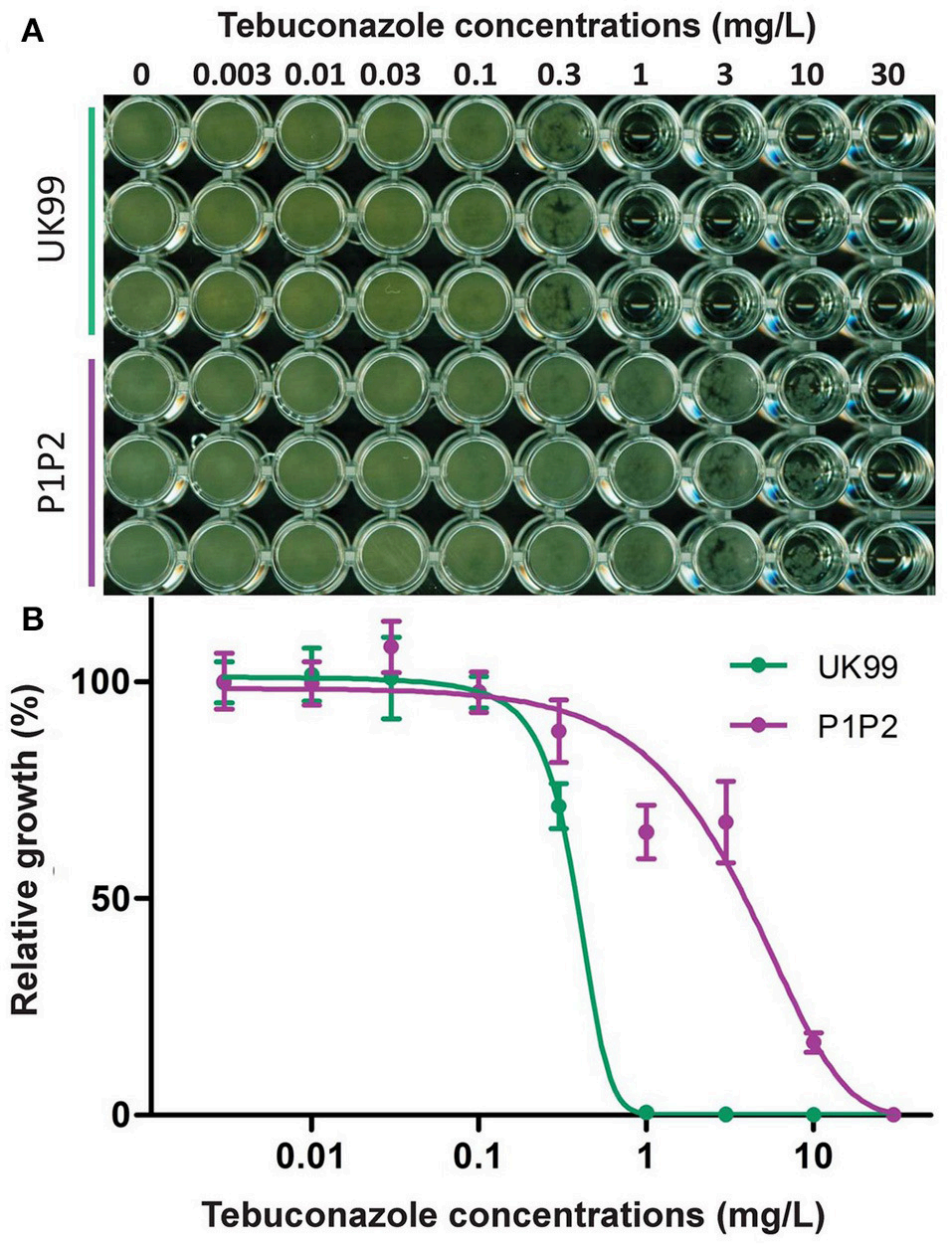
Comparison of tebuconazole sensitivity of the adapted strain (P1P2) with its parental strain (UK99) in a microtiter plate assay. **(A)** Strains inoculated in triplicates in potato dextrose broth amended with increasing concentrations of tebuconazole. **(B)** Absorbance readings (620 nm) were used to quantify fungicide sensitivity with a four-parameter logistic regression. Error bars represent the standard deviation between the means of three independent assays.

**Figure 3:**
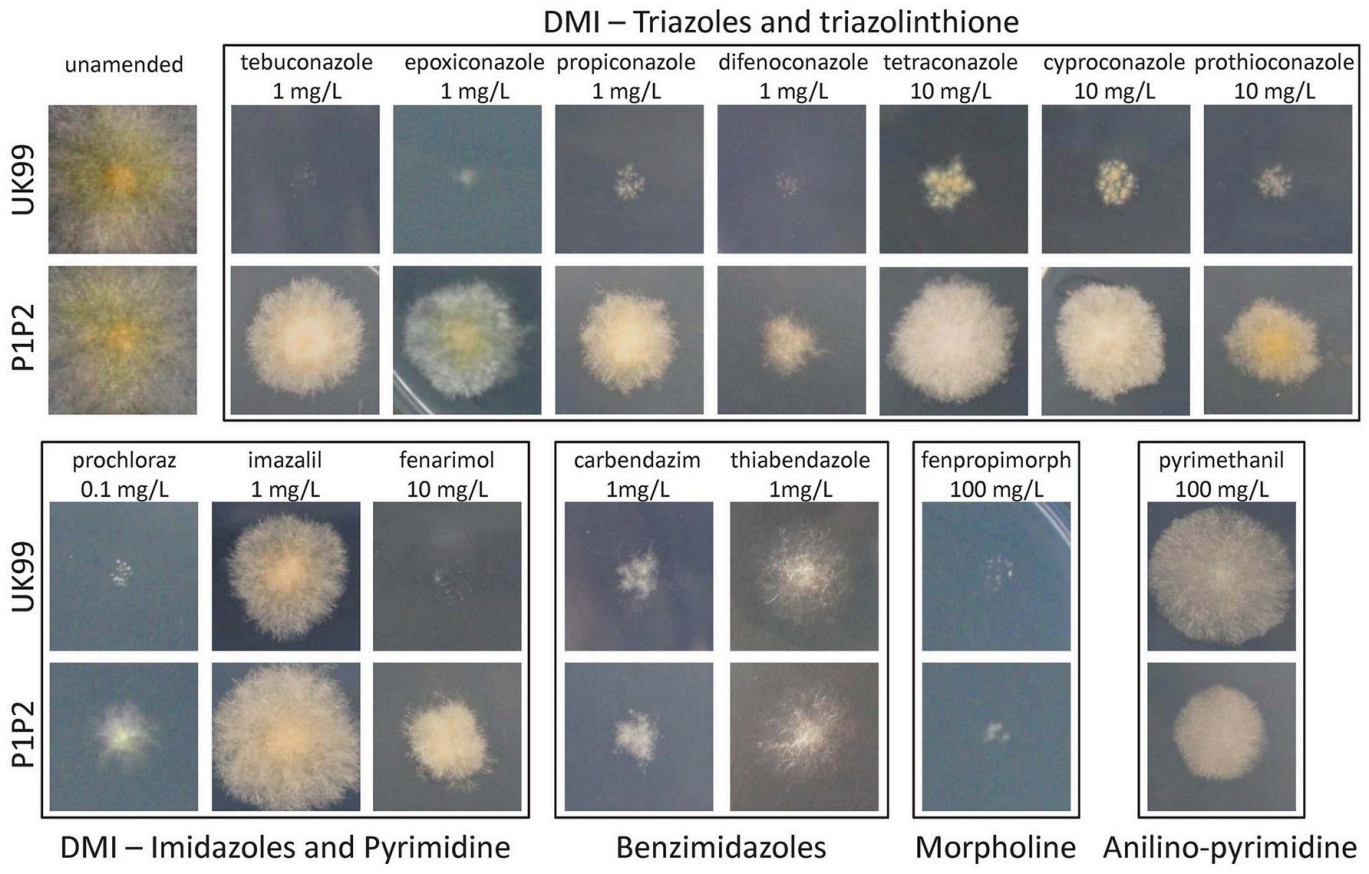
Qualitative cross-resistance comparison between UK99 and P1P2 to a variety of fungicides with different modes of action on PDA medium. Each image is a 2 × 2 cm square surrounding the inoculation point after 7 days of growth.

Strain P1P2 was found to be significantly more resistant than UK99 (*P* < 0.001, Figure 2). The EC_50_ value for P1P2 (3.63 mg/L ± 0.3) was found to be about 10-fold greater than for UK99 (0.37 mg/L ± 0.001). The acquisition of tebuconazole resistance was stable over time given that the sensitivity of the seventh generation (EC_50_ = 2.93 mg/L ± 0.53 and 0.37 mg/L ± 0.006 for P1P2 and UK99, respectively; *P* = 0.088) did not significantly differ from the first. Cross-resistance was observed for all the tested DMIs (triazoles: tebuconazole, epoxiconazole, propiconazole, difenoconazole, tetraconazole and cyproconazole; triazolinthiones: prothioconazole; imidazoles: prochloraz and imazalil; pyrimidines: fenarimol), with P1P2 being able to develop on PDA media supplemented with fungicide concentrations highly inhibitory to UK99 growth (Figure 3). No differences were observed between strains when cultivated on media containing benzimidazoles (carbendazim and thiabendazole) or morpholine (fenpropimorph). Interestingly, the anilino-pyrimidine fungicide tested (pyrimethanil) showed a weaker efficacy on the parental strain than on the adapted strain.

### Transcriptome analysis revealed major differential expression between the treated parental and adapted strains

The transcriptome sequencing of tebuconazole-treated strains UK99 and P1P2 (three libraries per strain) produced a total of about 535 million reads (about 90 million per library), of which around 96% passed the Illumina filtering step (Table S1). Raw sequencing data were submitted to the European Nucleotide Archive (ENA) under the accession ERP105089. On average, 85% of the reads could be mapped to the published genome of UK99 (Urban et al., 2016). About 88% of the 12,537 annotated genes were expressed in each library. High similarity of read abundance per genes was found among libraries with libraries belonging to the same strain clustering together (Figure S2). A differential gene expression analysis performed to determine how differently P1P2 responded to tebuconazole as compared to UK99, revealed that 4940 transcripts (39.4% of annotated genes) had significantly different level of expression (*q* < 0.05). Among those transcript, 649 were upregulated and 1214 were downregulated (fold-change [FC] > 2) in tebuconazole-treated P1P2 as compared to treated UK99. Gene ontology (GO) term enrichment analysis showed that those genes generally belonged in the same categories of “molecular function,” “cellular component,” and “biological process” with only a higher count for the downregulated genes (level 2; Figure S3). The differences of gene expression of treated P1P2 ranged from up to 576 fold increase (FCUL_10778) and 32 fold decrease (FCUL_05061, Table 1) as compared to treated UK99. All three *CYP51s* had significantly different levels of expression (*CYP51A*: FC = 0.80, *q* < 0.001; *CYP51B*: FC = 0.09, *q* = 0.038; *CYP51C*: FC = 0.11, *q* = 0.02) but the magnitude of the fold-change was relatively small. Of the 4940 differentially expressed among strain P1P2 and UK99, 1408 were also differentially expressed in *F. graminearum* PH-1 after a tebuconazole treatment (Becher et al., 2011) and 78 of them were mutually upregulated in both comparisons (Table 1 and Table S2). A total of 17 SNPs were found in the coding regions of P1P2 transcripts when compared to the reference UK99 genome with 12 of them resulting in amino acid changes (Table 2 and Table S3).

**Table 1:** Top 20 genes with the highest change of gene expression of P1P2 as compared to UK99 when subjected to tebuconazole.

**Fc gene ID^a^**	**Fg gene ID^b^**	**Old Fg gene ID^c^**	**Blast description**	**Log_2_(FC)^d^**	***q*-value**
**TOP 20 UPREGULATED GENES IN P1P2**					
FCUL_10778	FgramPH1_01t25277	FGSG_07642	Pentalenolactone D synthase	9.17	<0.001
FCUL_05462	FgramPH1_01t12719	FGSG_03372	Hypothetical protein	6.54	<0.001
FCUL_08942	FgramPH1_01t20959	FGSG_10990	Nonribosomal peptide synthetase	6.26	0.007
FCUL_06718	FgramPH1_01t15629	FGSG_04581	Transcription factor	5.86	<0.001
FCUL_11938	FgramPH1_01t27981	FGSG_09076	Hypothetical protein	5.66	0.007
**FCUL_06717**	FgramPH1_01t15627	FGSG_04580	ATP binding cassette transporter	5.05	<0.001
FCUL_03752	FgramPH1_01t06339	FGSG_02641	Glucose transporter rco-3	4.7	0.004
FCUL_06619	FgramPH1_01t15371	FGSG_12251	Hypothetical protein	4.65	0.01
**FCUL_11936**	FgramPH1_01t06405	FGSG_02672	Ent-kaurene oxidase	4.59	<0.001
FCUL_05234	FgramPH1_01t12209	FGSG_03164	Hypothetical protein	4.26	<0.001
FCUL_09162	FgramPH1_01t21537	FGSG_11228	GMC oxidoreductase	4.23	<0.001
FCUL_03997	FgramPH1_01t09381	FGSG_15034	Hypothetical protein	4.19	<0.001
FCUL_06808	FgramPH1_01t15839	FGSG_04667	α-ketoglutarate-dependent sulfonate dioxygenase	4.04	0.01
FCUL_12351	FgramPH1_01t15901	FGSG_16066	Hypothetical protein	4.01	<0.001
FCUL_05043	FgramPH1_01t11775	FGSG_16357	Phosphoethanolamine n-methyltransferase 3	3.97	<0.001
FCUL_06826	FgramPH1_01t15935	FGSG_16060	Alkanesulfonate monooxygenase	3.94	<0.001
FCUL_11949	FgramPH1_01t28003	FGSG_09066	Hydrophobin 3 precursor	3.77	<0.001
FCUL_12358	FgramPH1_01t15903	FGSG_11577	Hypothetical protein	3.74	<0.001
FCUL_08941	FgramPH1_01t20957	FGSG_10989	Short-chain dehydrogenase reductase	3.67	0.002
FCUL_12331	FgramPH1_01t08795	#N/A	Hypothetical protein	3.54	<0.001
**TOP 20 DOWNREGULATED GENES IN P1P2**					
FCUL_05061	FgramPH1_01t11819	FGSG_03001	Hypothetical protein	−5	<0.001
FCUL_11186	FgramPH1_01t26259	FGSG_15560	Hypothetical protein	−4.92	<0.001
FCUL_02780	FgramPH1_01t06451	FGSG_02694	Spherulin 1b partial	−4.74	<0.001
FCUL_06652	FgramPH1_01t15459	FGSG_04503	α/β hydrolase	−4.62	<0.001
FCUL_11523	FgramPH1_01t27023	FGSG_09463	Related to Rtm1p	−4.4	<0.001
FCUL_10992	FgramPH1_01t25819	FGSG_07852	Short-chain dehydrogenase reductase	−4.4	<0.001
FCUL_07989	FgramPH1_01t18803	FGSG_05807	Platelet-activating factor acetylhydrolase	−4.39	0.007
FCUL_05569	FgramPH1_01t12957	FGSG_12425	Hypothetical protein	−4.33	<0.001
FCUL_05879	FgramPH1_01t13679	FGSG_03775	Hypothetical protein	−4.31	<0.001
FCUL_09092	FgramPH1_01t21365	FGSG_11162	α/β hydrolase	−4.14	0.041
FCUL_02116	FgramPH1_01t04887	FGSG_02023	Hypothetical protein	−4.13	<0.001
FCUL_09505	FgramPH1_01t22379	FGSG_06479	Hypothetical protein	−4.12	<0.001
FCUL_05374	FgramPH1_01t12533	#N/A	Hypothetical protein	−4.08	<0.001
FCUL_06662	FgramPH1_01t15493	FGSG_04516	Kinase-like protein	−4.06	<0.001
FCUL_05450	FgramPH1_01t12693	#N/A	Transcriptional regulatory protein	−4.02	<0.001
FCUL_09693	FgramPH1_01t22845	FGSG_06656	Sphingoid long-chain base transporter rsb1	−4.01	<0.001
FCUL_03573	FgramPH1_01t08257	FGSG_10500	Pyridine nucleotide-disulfide oxidoreductase family	−3.99	<0.001
FCUL_03340	FgramPH1_01t07709	#N/A	Hypothetical protein	−3.97	<0.001
FCUL_06324	FgramPH1_01t14693	FGSG_04188	Related to major facilitator (MFS1) transporter	−3.95	<0.001
FCUL_08886	FgramPH1_01t20827	FGSG_10930	Hypothetical protein	−3.89	<0.001

Table sorted by Log_2_ fold change.

a Gene ID in the F. culmorum (UK99) genome annotation (Urban et al. 2016). Bold genes were also found to be upregulated in F. graminearum (PH-1) after a tebuconazole treatment (Becher et al., 2011).

b Gene id in the most recent version of F. graminearum (PH-1) genome annotation (King et al., 2015).

c Gene id in the previous version of F. graminearum (PH-1) genome annotation (Cuomo et al., 2007).

d *Fold change of expression between tebuconazole-treated P1P2 and UK99 strains*.

**Table 2:** Single nucleotide polymorphism (SNP) found in the coding regions of P1P2 transcripts when compared to the reference UK99 genome.

**Gene ID**	**Codon change^a^**	**Effect^b^**	**Blast description**
FCUL_00816	gaG/gaT	E7D	Small nuclear ribonucleoprotein
FCUL_02215	gGc/gAc	G450D	Feruloyl esterase b
FCUL_02467	cCa/cGa	P757R	Polyketide synthase
FCUL_03745	Cat/Gat	H240D	Unnamed protein product
FCUL_05178	atG/atC	M37I	Hypothetical protein
FCUL_05369	tTc/tCc	F716S	Transcription factor
FCUL_05892	gCt/gTt	A474V	Transcription factor
FCUL_06062	Cat/Gat	H185D	Heterokaryon incompatibility
FCUL_06890	Tct/Cct	S118P	Hypothetical protein
FCUL_06925	acT/acG	–	Endo-beta-glucanase
FCUL_09267	acC/acA	–	Hypothetical protein
FCUL_09409	gGg/gAg	G109E	Hypothetical protein
FCUL_10796	gaC/gaT	–	Hypothetical protein
FCUL_12334	gGt/gAt	G317D	Hypothetical protein
FCUL_12417	aGg/aAg	R213K	Hypothetical protein

a *Position of the SNP (in upper case) in the codon*.

b *Predicted amino acid change resulting from SNP*.

### Overexpression of *FcABC1* is confirmed in tebuconazole-treated field isolates

**Figure 4:**
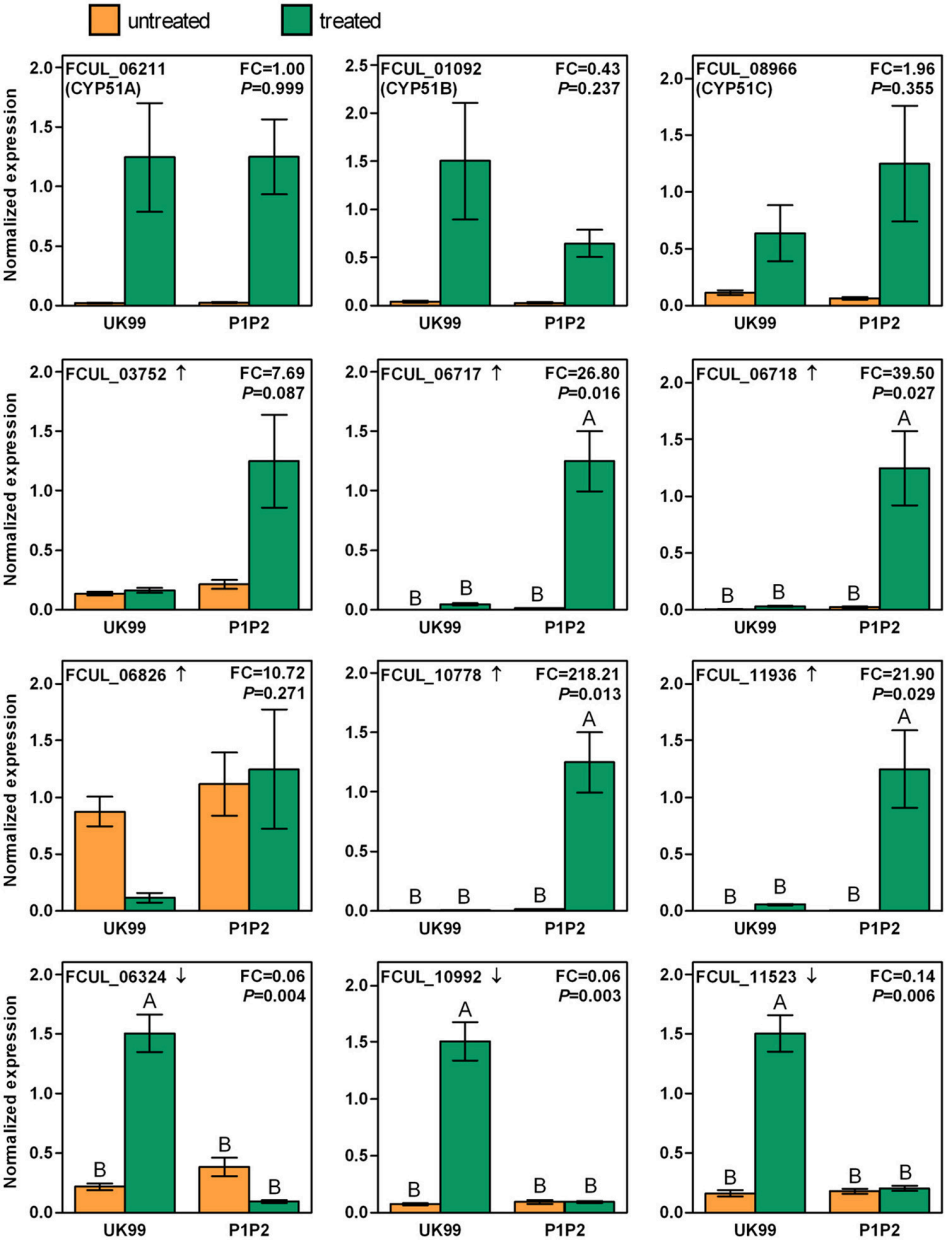
Expressions of selected genes in the resistant strain (P1P2) and in the wild-type strain (UK99) of *Fusarium culmorum* with (treated) or without (untreated) tebuconazole treatment (2.5 mg/L). The arrow next to the gene ID indicates whether the gene transcription was up or downregulated in the RNA-Seq experiment. “FC” reports the fold-change of expression measured between the P1P2 and UK99 when treated. The *P*-value represents the significance level of the interaction factor between strains and treatments in the ANOVA. Different letters on top of bars correspond to significantly different levels of expression determined by Tukey's test, performed when the interaction was significant. Error bars represent standard errors.

Among the most differentially expressed genes, the expression levels of a subset of interesting genes were investigated using RT-qPCR in order to validate the results of the RNA-Seq experiment and to compare the expression of the treated samples to that of the control samples (Figure 4). The detailed results of all statistical tests are presented in Table S4. The expression fold change measure for each gene in P1P2 and UK99 was of the same order of magnitude when comparing the results from the RNA-Seq experiment and the RT-qPCR assays (Figure S4). The expression levels of *CYP51A, B* and *C* were all significantly enhanced in both strains after treatment, but the slight significant downregulation between treated samples observed in the RNA-Seq experiment could not be confirmed by RT-qPCR (Figure 4). Among the selected upregulated genes, the expression levels of FCUL_06717, FCUL_06718, FCUL_10778, and FCUL_11936 were all significantly higher in P1P2 after a tebuconazole treatment than in any other samples. The expression pattern of FCUL_03752 followed the same pattern as the previous genes but the differences observed were not significant. No statistical difference was found among samples in the case of FCUL_06826. The three downregulated genes selected all presented a similar pattern of expression with the treated UK99 strain showing the highest expression levels.

**Figure 5:**
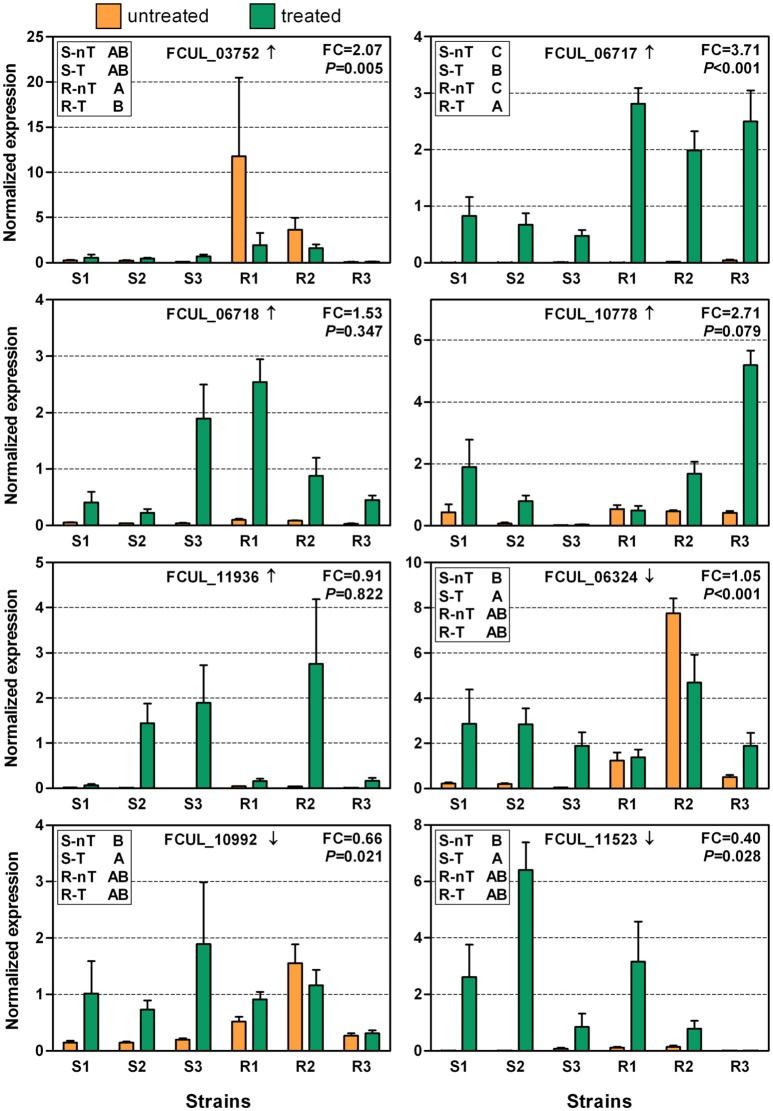
Expressions of selected genes in triazole-sensitive (S1 = MBC 6020, S2 = MBC 7603 and S3 = MBC 7964) and triazole-resistant (R1 = MBC 190, R2 = MBC 7555, and R3 = CRA PAV ER 1998) strains of *Fusarium culmorum* with (treated or T) or without (untreated or nT) tebuconazole treatment (2.5 mg/L). The arrow next to the gene ID indicates whether the gene transcription was up or downregulated in the RNA-Seq experiment. “FC” reports the fold-change of expression measured between the resistant and sensitive strains when treated. The *P*-value represents the significance level of the interaction factor between resistance levels and treatments in the linear mixed model. In the top left frame, group (e.g., S-nT) with different letters associated to them have significantly different levels of expression as determined by Tukey's test, performed when the interaction was significant. Error bars represent standard errors.

The expression of all of these genes, except for *CYP51s* and FCUL_06826, was subsequently analyzed on a set of *F. culmorum* strains considered to be either triazole-sensitive or resistant (Figure 5). Overall, the gene expression levels appeared to be more consistent and homogenous among the sensitive strains than among the resistant strains and differences between the control and the treated samples were therefore usually significant for the sensitive strains. As for UK99 (Figure 4), the sensitive strains generally responded with an upregulation of the tested genes after treatment, whereas it was not always the case for the resistant strains. For instance, the expression pattern of FCUL_06324 was significantly upregulated in UK99 after treatment (Figure 4) and it was also the case for all sensitive strains (Figure 5). Yet, the same gene was slightly downregulated in P1P2 after treatment (Figure 4) but its expression was highly variable among resistant strains, resulting in no overall statistical differences between control and treated samples (Figure 5). The expression pattern of FCUL_06717, characterized by a higher overexpression of the transcript in the resistant strain than in the sensitive strain, was the only one that was consistent with the pattern observed for both P1P2 and UK99 (Figure 4). Furthermore, statistical analysis using Tukey's test confirmed that the resistant strains confronted to tebuconazole had the highest level of expression for FCUL_06717, followed by the treated sensitive strains whereas the expression of the gene in the untreated controls in both groups of strains were equally lower (Figure 5). However, the average expression fold-change observed when comparing the treated resistant strains to the treated sensitive strains (*FC* = 3.71; Figure 5) was considerably lower than between P1P2 and UK99 (*FC* = 26.8; Figure 4). The pattern of expression of other genes (FCUL_03754, FCUL_06718, FCUL_10778, and FCUL_11936) did not support their consistent involvement in the resistance naturally occurring in the field (Figure 5).

### Resistance is not the result of a sequence modification in *FcABC1*

*FcABC1* and its flanking regions were sequenced for the adapted and parental strains in order to identify potential modifications that might have occurred in the gene's regulatory regions that would be responsible of the higher level of observed after azole treatment in the adapted strain. No differences were found in the 415 bp region upstream of the start codon of *FcABC1* neither between the two strains nor among the field isolates. The 448 nucleotides sequenced downstream of the stop codon, did not reveal any differences either between P1P2 and UK99. The gene itself was 4,569 bp long compared with 4,580 bp and 4,584 bp in *F. graminearum* (FGSG_04580) and *F. pseudograminearum* (Aoki & O'Donnell) (FSPE_06011). The sequence of UK99 had only one nucleotide difference with the one of *F. culmorum* CS7071 at position 3090. This mutation at the third position of the codon between C (UK99) and T (CS7071), is however silent, leading to a leucine at amino acid position 1030. Nevertheless, there was no amino acid difference between the parental and adapted strains in this region. All three sequences for UK99/P1P2 were stored in GenBank under Accession No. KX601158. Phylogeny showed that FcABC1 has homologs in multiple other fungal species/genera (Figure S5).

## Discussion

The baseline sensitivity of *F. culmorum* to triazoles has been previously shown to be quite stable over time, although more resistant strains could be observed in the field. As a result, it has been postulated that the acquisition of resistance could come at a cost preventing the development of a resistant population in the field (Hellin et al., 2016b). Indeed, an *in vitro* fitness penalty had been observed by Serfling and Ordon (2014) for a *F. culmorum* strain adapted to tebuconazole. In order to verify this hypothesis an adaptation method, inspired by previous methodologies (Becher et al., 2010; Serfling and Ordon, 2014), was performed on the sensitive strain UK99. This method had the advantage of bypassing the use of mutation inducers, such as UV light, that introduce random mutation in the genome. Instead, it applied a tebuconazole-specific selective pressure on the strain that better mimicked what would naturally occur in the field. Remarkably, this procedure allowed producing and selecting a *F. culmorum* strain (P1P2) that was not only more resistant to tebuconazole than its parental strain, but with almost no other phenotypic changes. Indeed, all fitness parameters tested (sporulation, growth and pathogenicity) were practically identical between the two strains. Moreover, in *vitro* experiments showed that not only was that strain more resistant to tebuconazole (by about 10-fold) and every other tested DMI, but also that the acquired resistance was specific to this fungicide group. Their common mode of action enable fungi to develop cross-resistance to these molecules and this phenomenon has been often reported for *Fusarium* spp. (Yin et al., 2009; Becher et al., 2010; Serfling and Ordon, 2014; Spolti et al., 2014) as well as for other fungi such as *Ustilago maydis* (DC.) Corda, *Sclerotinia homoeocarpa* (F.T. Bennett), *Rhynchosporium secalis* ([Oudem.] Davis) and *Zymoseptoria tritici* ([Desm.] Quaedvlieg & Crous) (Wellmann et al., 1996; Hsiang et al., 1997; Robbertse et al., 2001; Cools and Fraaije, 2012). The adapted strain even seemed to be a little less affected by a curative tebuconazole treatment applied on infected wheat ears compared with the wild-type strain, although this difference was not statistically supported. In their attempt to adapt *in vitro* an *F. culmorum* strain to azoles, Serfling and Ordon (2014) produced a strain that had a reduced *in vitro* fitness, but was insensitive to azole treatment *in planta* compared with its parental strain. Taking together, these observations supports the possibility that the repetitive use of triazoles in the field could naturally give rise to competitive *F. culmorum* strains with triazole resistance.

Taking advantage of the similar genetic background of UK99 and P1P2, we compared their transcriptome after tebuconazole treatment to detected targets with a potential role in DMI resistance. Surprisingly, the transcriptomic differences observed between the 2 treated strains were drastically different with significant differences observed for almost 40% of the genes in the currently annotated version of *F. culmorum* genome (Urban et al., 2016). Surprisingly, this difference in gene expression was even higher than what was observed for *F. graminearum* PH-1 treated or not with tebuconazole (Becher et al., 2011). Several authors have used different genome wide approaches to detect gene targets involved in *F. graminearum* response or sensitivity to triazoles (Liu et al., 2010; Becher et al., 2011; Talas et al., 2016). Interestingly, none of the genes highlighted in their study were present among the most differentially regulated genes (with homologs in *F. graminearum*) between the treated *F. culmorum* strains UK99 and P1P2, indicating the complementarity of the studies.

Only 12 non-synonymous mutations were detected in the coding regions of P1P2 genes as compared to the reference *F. culmorum* genome and none of them occurred in the *CYP51* genes. In a genome-wide association study, Talas et al. (2016) detected 51 genes with SNPs that could be linked to propiconazole sensitivity over 220 strains of *F. graminearum*. None of those SNPs occurred in the CYP51 genes nor were common with the SNPs in *F. culmorum* homologs observed in the present study. Moreover, no nucleotide changes leading to amino acid substitutions were found in the CYP51B gene of the more resistant *F. culmorum* field isolates investigated in this study (data not shown). Adding to the fact that mutations in CYP51*s* were not linked to the azole sensitivity of *F. graminearum* field isolates in other studies (Yin et al., 2009; Talas and McDonald, 2015) and that mutations in CYP51s might only be associated with resistance to a subset of DMI fungicides as observed for *Z. tritici* (Cools and Fraaije, 2012), these results suggest that mutations in CYP51s do not play a role in the resistance of the DMI-adapted *F. culmorum* strain P1P2.

The overexpression of CYP51 genes is a common resistance mechanism that has been reported for numerous species (Lucas et al., 2015; Ziogas and Malandrakis, 2015) and can yield to pan-azole resistant phenotypes (Cools et al., 2012). Indeed, the expression of all *CYP51s* was increased after the tebuconazole treatment. Although, this overexpression was found to be significantly different between the two strains in the RNA-Seq, the fold-change was lower than two and the increase was always in favor of the sensitive strain UK99. Moreover, qPCR analysis did not confirm this difference. Other studies on *F. graminearum* have also highlighted the upregulation of the *CYP51* genes after a triazole treatment (Yin et al., 2009; Liu et al., 2010; Becher et al., 2011) but no relation with the level of triazole sensitivity could be observed (Yin et al., 2009). Therefore, the upregulation of sterol biosynthetic genes is not likely to be a part of the mechanism developed by P1P2 to resist to DMI fungicides.

After the investigation of the expression profiles of multiple genes that were significantly more expressed in the resistant strain P1P2 upon treatment, *FcABC1* (FCUL_06717) appeared as a suitable candidate to explain the difference in DMI sensitivity between the two strains. Moreover, this gene was also shown to be upregulated in *F. graminearum* after a tebuconazole treatment (Becher et al., 2011). The gene demonstrated an expression level about 30-fold higher in P1P2 than the in parental strain after tebuconazole treatment. This difference in expression could be verified on field strains harboring distinct azole sensitivity levels, with the resistant strains showing a higher overexpression of the transporter than the sensitive strains upon tebuconazole treatment. Overexpression of a transporter is a common feature developed by fungi in response to triazole pressure (Ziogas and Malandrakis, 2015). For example, in *S. homoeocarpa*, the pathogen causing dollar spot on turf grass, the overexpression of two ABC transporters, ShPDR1 and ShatrD, was linked to practical field resistance (Hulvey et al., 2012; Sang et al., 2015). The modulation of transporter expression can be due to mutations or indels occurring in the gene's regulatory regions. A 519 bp insert has, for example, been linked with the overexpression of an MFS (Major facilitator superfamily) transporter conferring multidrug resistance in *Z. tritici* (Omrane et al., 2015). Yet, no difference could be found in the upstream region of *FcABC1*, neither in the gene itself nor in the 448 bp following its stop codon, suggesting that another regulatory mechanism might be involved, such as overexpression of a transcription factor or even an epigenetic modification. Interestingly, a zinc finger transcription factor (FCUL_06718) located next to *FcABC1* in the genome of UK99, showed the same difference of expression as *FcABC1* between the parental and resistant strain. Unfortunately this pattern of expression was not completely matched by the expression profile observed for in the resistant and sensitive field strains.

The protein sequence of FcABC1 showed high homology with multiple proteins present in different species of *Fusarium*, including *F. graminearum* (i.e., FgABC3) in which it has been shown to be implicated in azole sensitivity (Abou Ammar et al., 2013), as well as in other genera such as *Trichoderma* and *Colletotrichum*. Therefore, it can be anticipated that overexpression of these homologous proteins could enhance resistance in the corresponding species. In line with our findings, the sensitivity levels to the various DMI fungicides of *F. graminearum* mutants lacking FgABC3 were shown to increase while no change in sensitivity were observed with regard to selected strobilurin, amines, SDHI (succinate deshydrogenase inhibitors), quinone, N-phenyl carbamate or thiocarbamate (Abou Ammar et al., 2013). However, *FgABC3* mutants produced by Gardiner et al. (2013) were found to be more sensitive to benalaxyl, a phenylamide fungicide targeting RNA polymerase I, for which the resistance mechanism remains unknown (FRAC, 2018). In the present study, the resistant strain was found to be less sensitive to pyrimethanil, an anilino-pyrimidine fungicide interfering with methionine biosynthesis (FRAC, 2018). This kind of negative cross-resistance among active ingredients has been reported in some drug/microbe systems (Pál et al., 2015). Functional analysis of FcABC1 by gene disruption had previously revealed its role in wheat and barley infection by *F. culmorum* (Skov et al., 2004). Similarly, *F. graminearum* mutants lacking the *FcABC1* homolog (*FgABC3*) showed a reduced virulence on wheat altough the growth rate of *FgABC3* mutants subjected to various defensive plant metabolites appeared to be unaffected (Abou Ammar et al., 2013; Gardiner et al., 2013). Interestingly, in *F. sambucinum* (Fuckel), the FcABC1 homologous transporter is necessary to protect the pathogen against rishitin, a phytoalexin present in potato tubers (Fleissner et al., 2002). As it would be expected from PDR transporters, the specificity of FcABC1 and homologs might therefore not be restricted to a single chemical family such as DMI fungicides.

The field strains used in this study exhibited highly contrasted sensitivity levels to triazoles but it has been shown that DMI fungicides usually give rise to a more continuous distribution of resistance degree in the field (Lucas et al., 2015). Therefore, the implication of FcABC1 in triazole resistance might only be linked to a particular phenotype of highly resistant strains and other processes could be involved in *F. culmorum* resistance, as it has been seen in other fungi such as *Z. tritici* (Cools and Fraaije, 2012). As fungal populations evolve, among other things, in response to fungicide pressure, it is possible that different types of mechanisms will develop in the future. Detoxification of the fungicide molecules is the less frequently reported resistance mechanism. In this study, FCUL_06826, FCUL_10778, and FCUL_11936, enzymes with potential degradation capacity were investigated but their implication in DMI resistance was not confirmed in *F. culmorum* field strains, even though the homolog of FCUL_11936 has also been shown to be upregulated in *F. graminearum* upon tebuconazole treatment (Becher et al., 2011). Mechanisms of detoxification of drugs are well known in herbicide resistance and usually involve enzymes such as cytochrome P450 monooxygenase, glycosyl transferase and glutathione *S*-transferase (Yu and Powles, 2014). Only a few cases of resistance by fungicide degradation have been reported such as the degradation of kresoxym-methyl by an esterase in *Venturia inequalis* (Jabs et al., 2001) or of fenhexamid by a cytochrome P450 monooxygenase in *Botrytis cinerea* (Leroux et al., 2002).

Understanding the current and future challenges of resistance development is mandatory to preserve the field efficacy of triazoles in the management of *Fusarium* spp. related diseases and the discovery of molecular mechanisms underlying resistance will improve the management strategies. Ultimately, new targets for the chemical control arise from mechanistic studies and might one day lead to the development of new fungicides that circumvent the existing resistance.

## Data availability

The datasets for this study are available on request.

## Author contributions

PH and AL contributed conception and design of the study. PH performed most of the experiments and analysis. RK analyzed the RNA-Seq data. PH and AL wrote the first draft of the manuscript. RK, MU, and KH-K wrote sections of the manuscript. All authors contributed to the revision of the manuscript, read and approved the submitted version.

### Conflict of interest statement

The authors declare that the research was conducted in the absence of any commercial or financial relationships that could be construed as a potential conflict of interest.

## Supplementary material

Figure S1 Summary of the screening procedure to select a triazole-adapted phenotype from *F. culmorum* strain UK99. **(A)** Conidia solutions point-inoculated on PDA amended with 1 mg/l of tebuconazole incubated for 4 days. **(B)** Sensitivity (EC50) of the strains to tebuconazole measured by microtiter plate assays **(C)** Growth on PDA after 4 days. **(D)** Pathogenicity to wheat seedlings grown on SNA in test tubes measured by growth retardation and symptom severity. **(E)** Pathogenicity to wheat ears of point-inoculated conidia suspension with or without a curative tebuconazole treatment. Error bars are SEM.

Figure S2 Comparison of transcript abundances among each library. **(A)** Heat map showing the hierarchical clustering (complete linkage method) of the Euclidian distance between transcript abundance of each library. **(B)** Score plot of the principal component analysis performed on the transcript abundance matrix of all libraries.

Figure S3 Functionnal characterization of the differentially genes expressed (FC >2 and *q* < 0.05) in the treated resistant strain (P1P2) as compared to its treated parental strain (UK99). The gene onthology terms (level 2) for the 469 up- and 1214 down-regulated genes plotted are sorted into three categories for biological processes, cellular component and molecular function.

Figure S4 Expression fold-change of select genes between the resistant strain (P1P2) and the sensitive strain (UK99) both treated with tebuconazole in the RNA-Seq experiment as compared to the RT-qPCR assays.

Figure S5 Neighbor joining phylogenetic tree representing evolutionary relationships between *F. culmorum* ABC1 and other selected ABC transporters protein sequences (*N* = 34). Entries in bold correspond to ABC transporters known for their implication in demethylation inhibitor (DMI) resistance in other plant pathogenic fungi. Sequences from *F. graminearum* are in red. Sequences were aligned using MUSCLE and phylogenetic relationships were then calculated using the neighbor joining method with the default parameters implemented in MEGA6. Branch support, obtained with 1,000 bootstrap iterations, is indicated by the relative size of the black dot on the corresponding branch.

Table S1 Metrics summarizing the transcriptome sequencing and alignment to UK99 genome of each library (3) belonging to the tebuconazole-treated strains of UK99 and P1P2.

Table S2 Detailed list of all transcripts mutually upregulated both in this study (treated-Fc_P1P2 vs treated-Fc_UK99) and in Becher et al. (2011) (treated-Fg_PH1 vs untreated-Fg_PH1).

Table S3 Detailed list of all single nucleotide polymorphisms (SNPs) found when comparing P1P2 transcriptome to the reference UK99 genome.

Table S4 Statistical significance levels (*P*-values) of the parameters used to explain gene expression differences observed in the RT-qPCR experiments.

Table S5 List primers used for quantification of transcript level of candidate genes.

Table S6 List of primers used for amplification and sequencing of FcABC1.
