# Supplementary figures and images for: The adaptation of Fusarium culmorum to DMI Fungicides Is Mediated by Major Transcriptome Modifications in Response to Azole Fungicide, Including the Overexpression of a PDR Transporter (FcABC1)
Source: Front Microbiol. 2018 Jun 26;9:1385. doi: 10.3389/fmicb.2018.01385 (PMC6028722; doi:10.3389/fmicb.2018.01385)

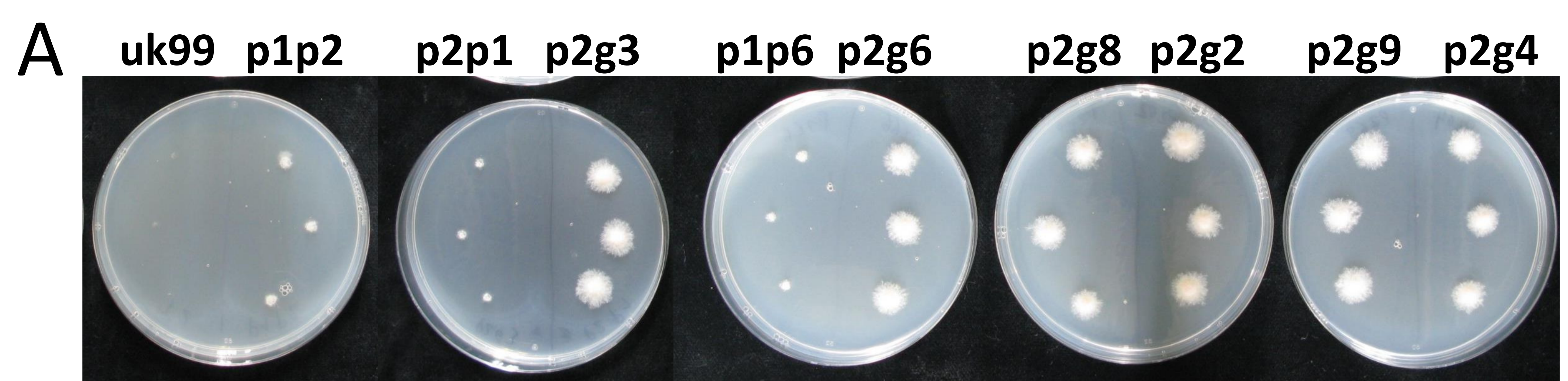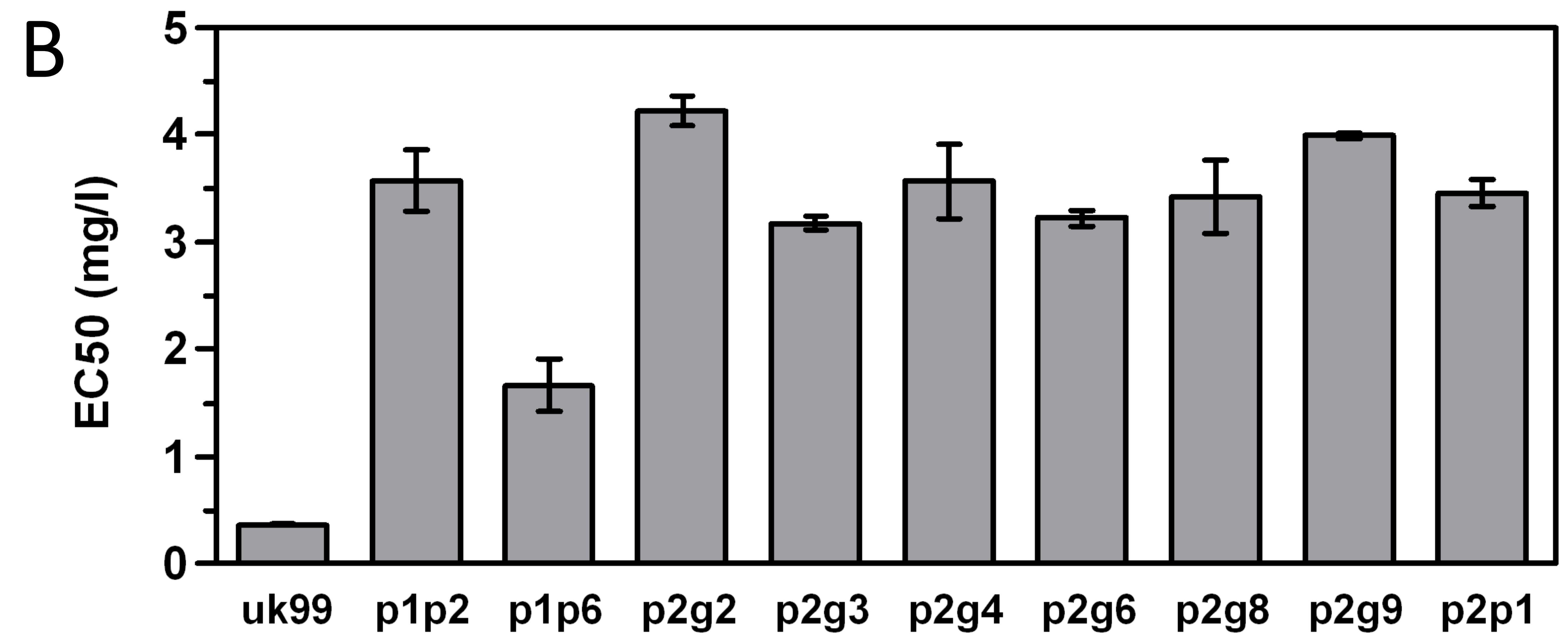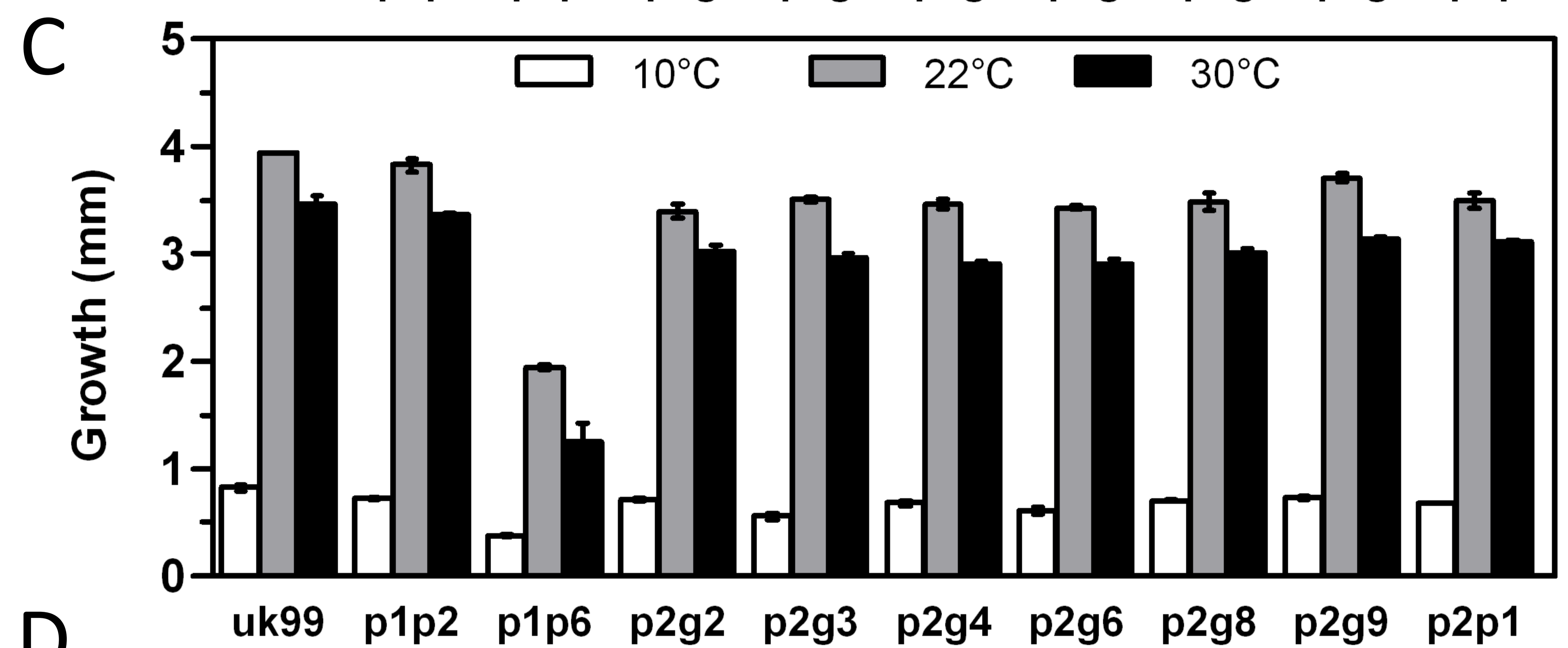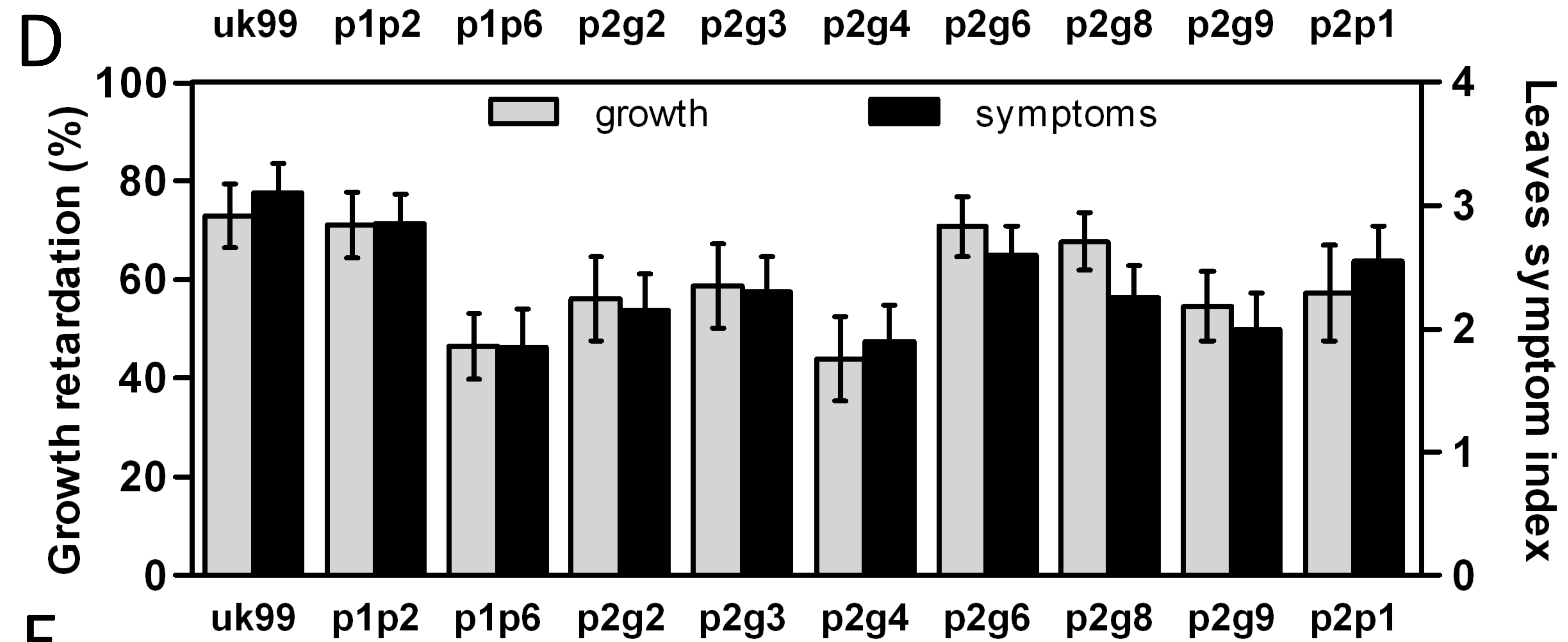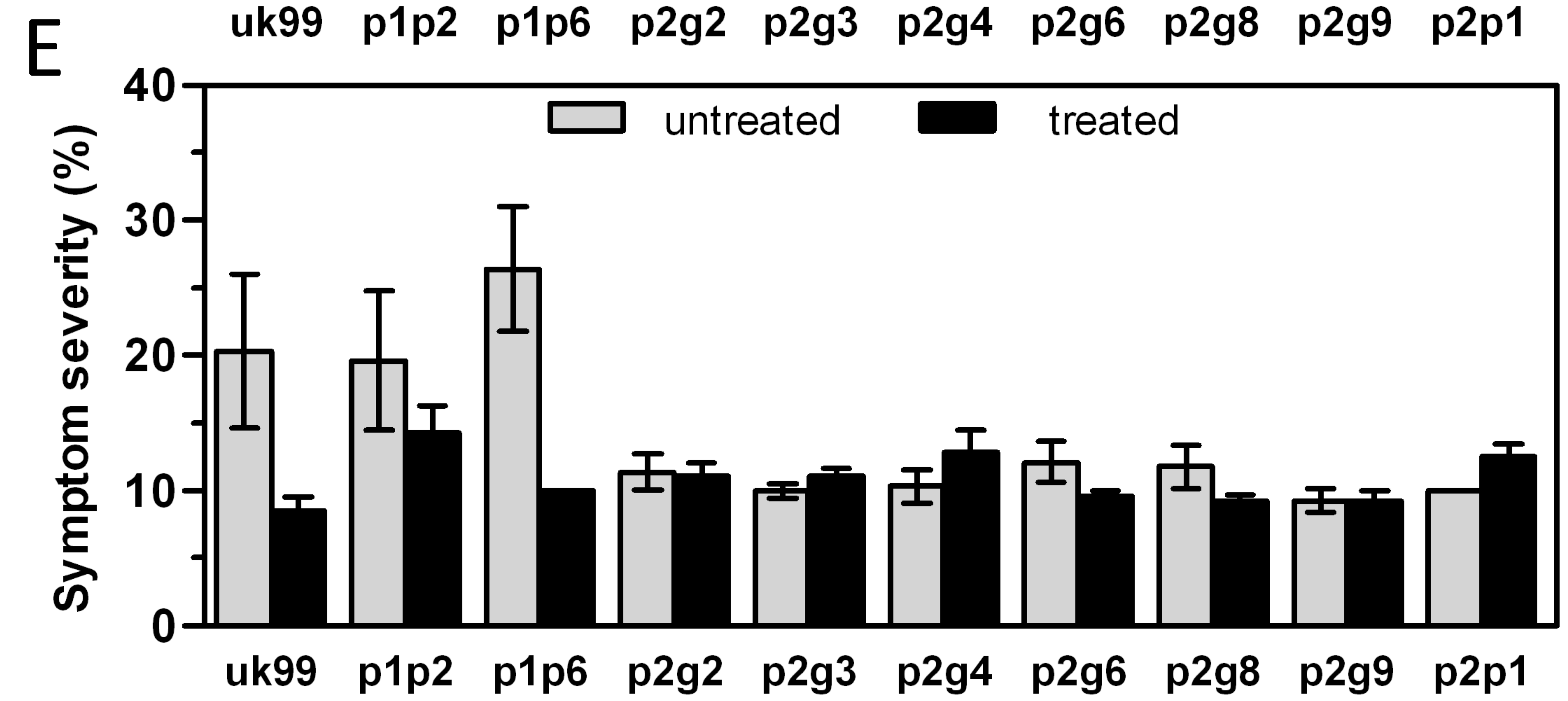

Supplement: Figure S1 — Summary of the screening procedure to select a triazole-adapted phenotype from F. culmorum strain UK99. (A) Conidia solutions point-inoculated on PDA amended with 1 mg/l of tebuconazole incubated for 4 days. (B) Sensitivity (EC50) of the strains to tebuconazole measured by microtiter plate assays (C) Growth on PDA after 4 days. (D) Pathogenicity to wheat seedlings grown on SNA in test tubes measured by growth retardation and symptom severity. (E) Pathogenicity to wheat ears of point-inoculated conidia suspension with or without a curative tebuconazole treatment. Error bars are SEM. [file Image_1.PDF]

A

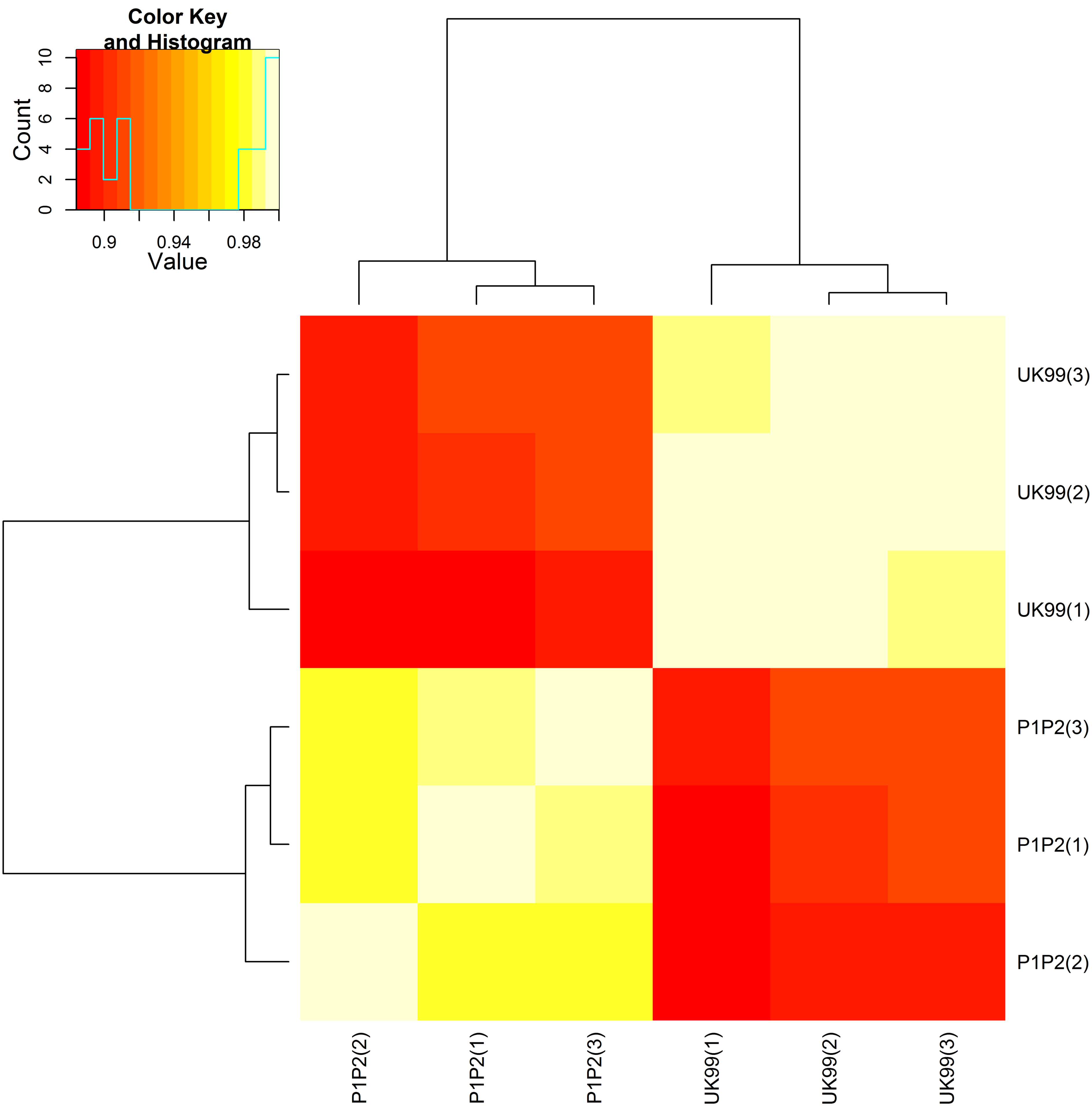

B

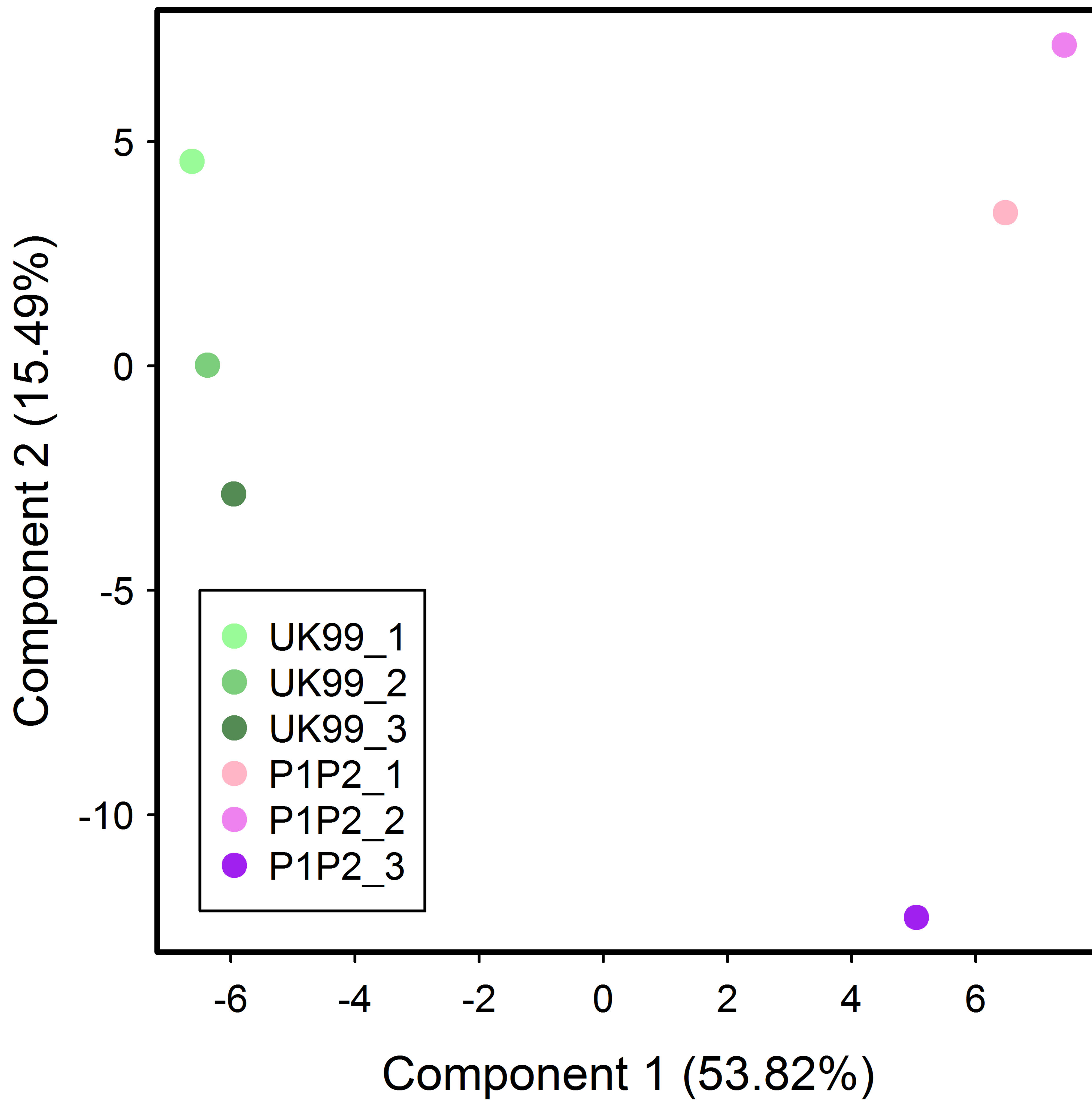

Supplement: Figure S2 — Comparison of transcript abundances among each library. (A) Heat map showing the hierarchical clustering (complete linkage method) of the Euclidian distance between transcript abundance of each library. (B) Score plot of the principal component analysis performed on the transcript abundance matrix of all libraries. [file Image_2.PDF]

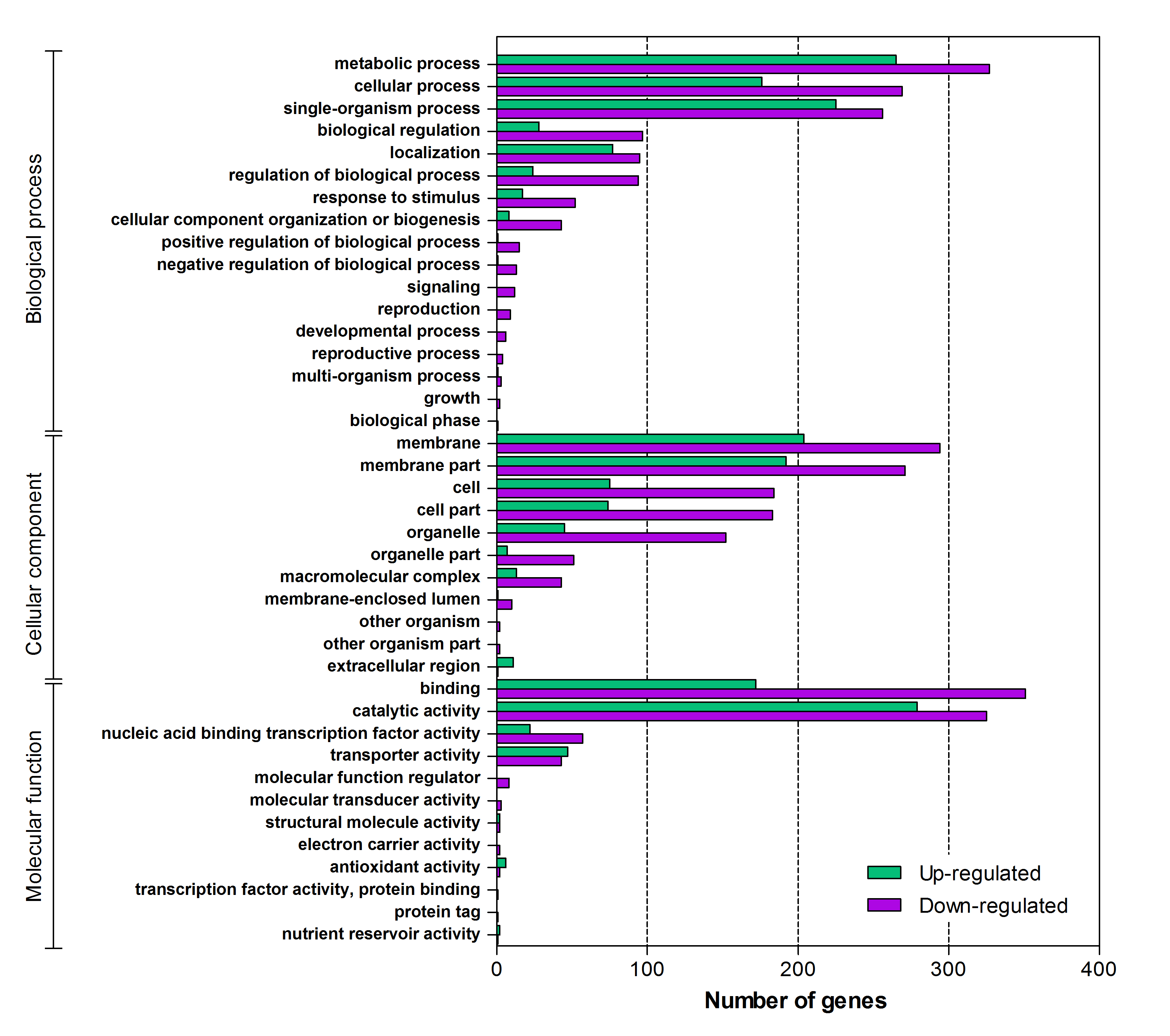

Supplement: Figure S3 — Functionnal characterization of the differentially genes expressed (FC >2 and q < 0.05) in the treated resistant strain (P1P2) as compared to its treated parental strain (UK99). The gene onthology terms (level 2) for the 469 up- and 1214 down-regulated genes plotted are sorted into three categories for biological processes, cellular component and molecular function. [file Image_3.TIF]

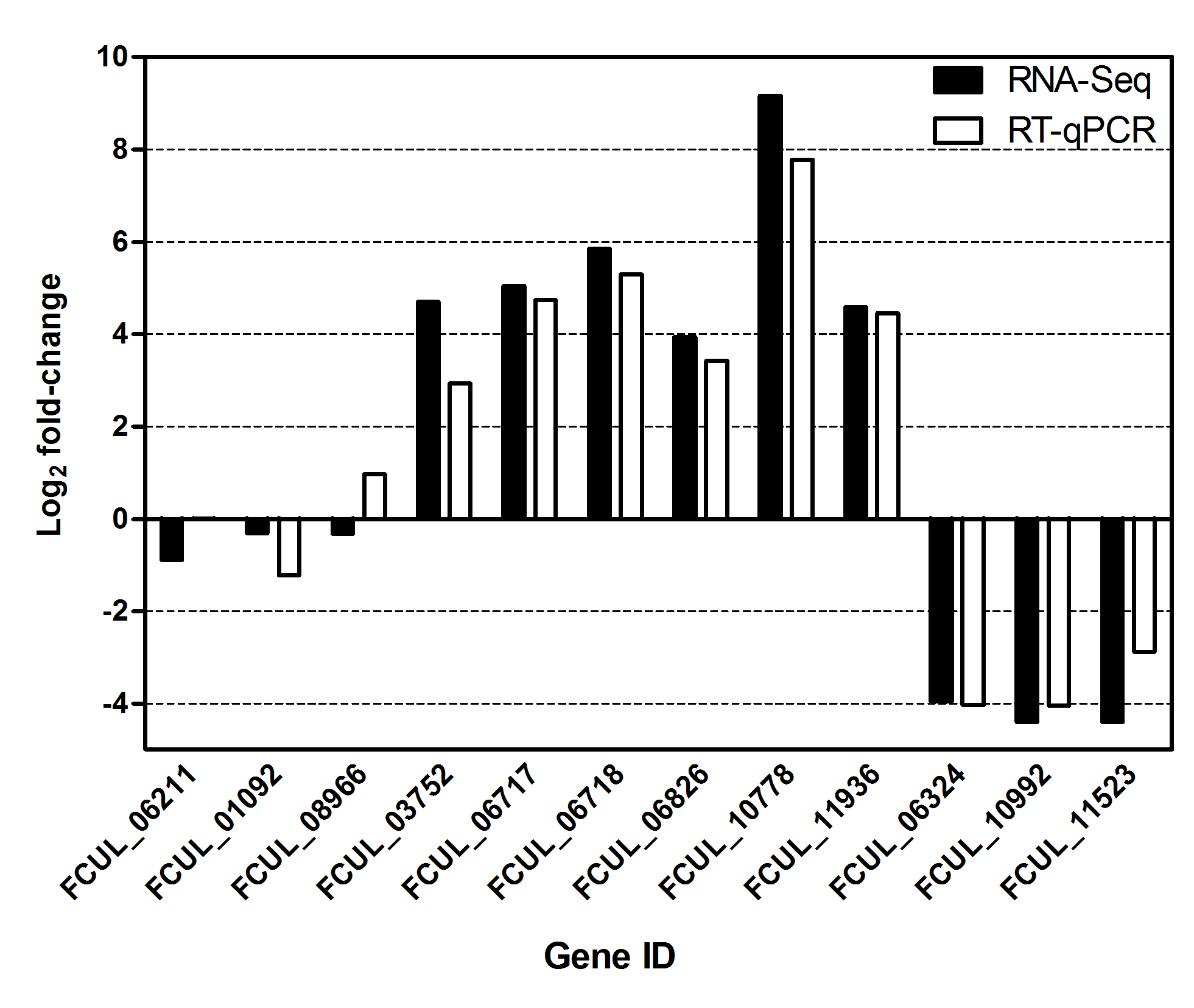

Supplement: Figure S4 — Expression fold-change of select genes between the resistant strain (P1P2) and the sensitive strain (UK99) both treated with tebuconazole in the RNA-Seq experiment as compared to the RT-qPCR assays. [file Image_4.TIF]

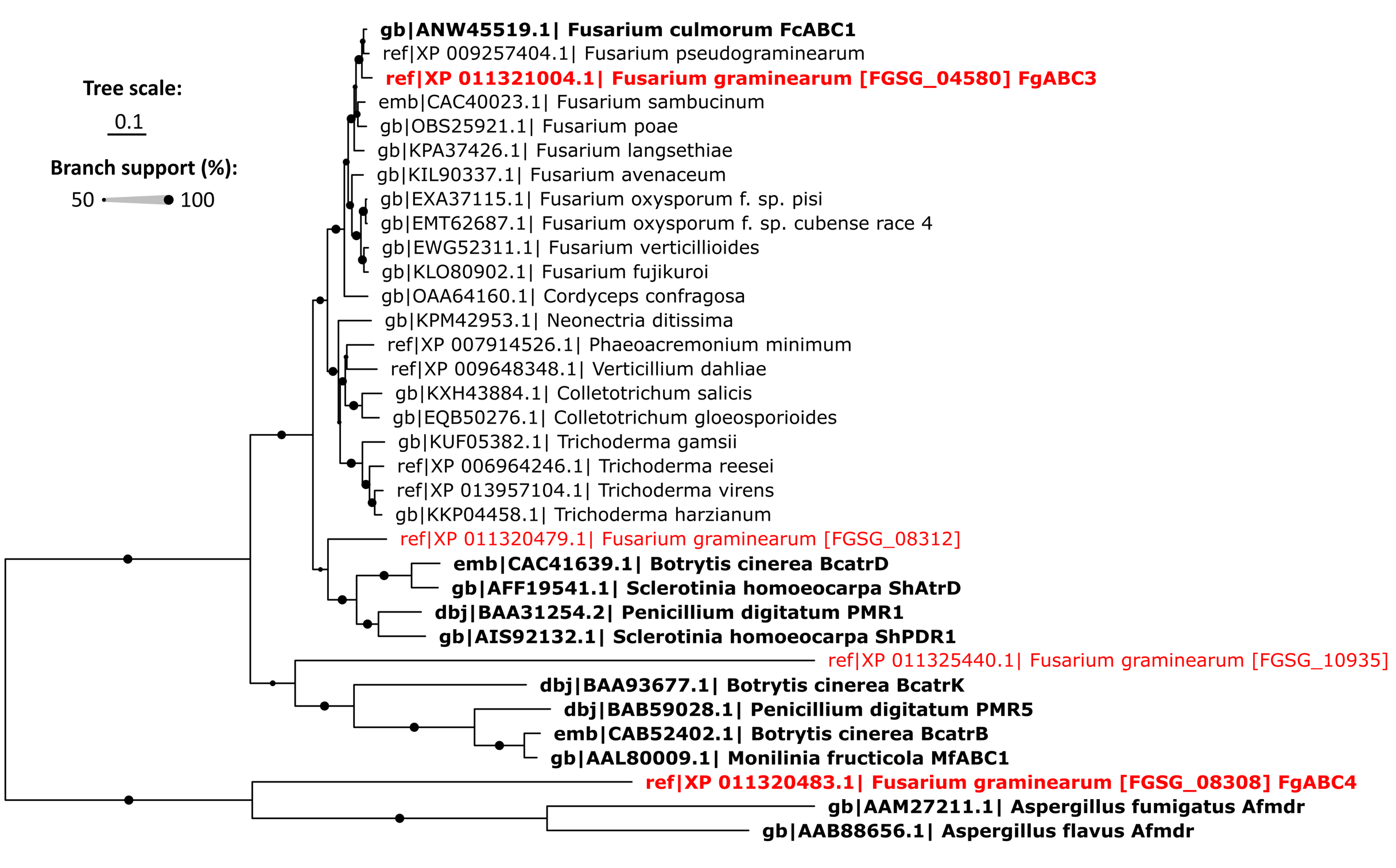

Supplement: Figure S5 — Neighbor joining phylogenetic tree representing evolutionary relationships between F. culmorum ABC1 and other selected ABC transporters protein sequences (N = 34). Entries in bold correspond to ABC transporters known for their implication in demethylation inhibitor (DMI) resistance in other plant pathogenic fungi. Sequences from F. graminearum are in red. Sequences were aligned using MUSCLE and phylogenetic relationships were then calculated using the neighbor joining method with the default parameters implemented in MEGA6. Branch support, obtained with 1,000 bootstrap iterations, is indicated by the relative size of the black dot on the corresponding branch. [file Image_5.PDF]
