# Supplementary material for: The adaptation of Fusarium culmorum to DMI Fungicides Is Mediated by Major Transcriptome Modifications in Response to Azole Fungicide, Including the Overexpression of a PDR Transporter (FcABC1)
Source: Front Microbiol. 2018 Jun 26;9:1385. doi: 10.3389/fmicb.2018.01385 (PMC6028722; doi:10.3389/fmicb.2018.01385)
Supplement: Table S1 — Metrics summarizing the transcriptome sequencing and alignment to UK99 genome of each library (3) belonging to the tebuconazole-treated strains of UK99 and P1P2. [file Table_1.PDF]

Table S1. Metrics summarizing the transcriptome sequencing and alignment to UK99 genome of each library (3) belonging to the tebuconazole-treated strains of UK99 and P1P2

	UK99						P1P2					
	1		2		3		1		2		3	
	Count	%	Count	%	Count	%	Count	%	Count	%	Count	%
Total reads	87,513,762	100	90,792,210	100	83,772,958	100	90,321,846	100	95,966,452	100	90,582,852	100
Passed filter	84,194,128	96.21	86,902,424	95.72	80,200,986	95.74	86,991,398	96.31	92,263,094	96.14	86,951,488	95.99
Read pairs	42,097,064	100	43,451,212	100	40,100,493	100	43,495,699	100	46,131,547	100	43,475,744	100
aligned concordantly once	20,308,498	48.24	18,869,704	43.43	18,796,940	46.87	21,429,937	49.27	22,004,905	47.7	21,140,375	48.63
aligned concordantly >1	10,822,487	25.71	13,538,527	31.16	11,092,929	27.66	11,176,651	25.7	11,919,199	25.84	11,601,389	26.68
aligned discordantly	2,698,166	6.41	2,287,569	5.26	2,454,861	6.12	2,735,745	6.29	3,128,005	6.78	2,727,757	6.27
aligned concordantly or discordantly 0 times	8,267,913	19.64	8,755,412	20.15	7,755,763	19.34	8,153,366	18.75	9,079,438	19.68	8,006,223	18.42
Unaligned reads	13,035,808	15.48	13,327,495	15.34	11,508,557	14.35	13,083,004	15.04	14,647,090	15.88	12,433,461	14.3
Aligned reads	71,158,320	84.52	73,574,929	84.66	68,692,429	85.65	73,908,394	84.96	77,616,004	84.12	74,518,027	85.7
