# Supplementary material for: The adaptation of Fusarium culmorum to DMI Fungicides Is Mediated by Major Transcriptome Modifications in Response to Azole Fungicide, Including the Overexpression of a PDR Transporter (FcABC1)
Source: Front Microbiol. 2018 Jun 26;9:1385. doi: 10.3389/fmicb.2018.01385 (PMC6028722; doi:10.3389/fmicb.2018.01385)
Supplement: Table S4 — Statistical significance levels (P-values) of the parameters used to explain gene expression differences observed in the RT-qPCR experiments. [file Table_4.PDF]

Table S4. Statistical significance levels (*P*-values) of the parameters used to explain gene expression differences observed in the RT-qPCR experiments.

Gene id	P1P2 vs UK99 [a]			Field strains [b]		
	Statistical significance by factor			Statistical significance by factor		
	Strain (S)	Treatment (T)	(S) x (T)	Resistance (R)	Treatment (T)	(R) x (T)
CYP51						
FCUL_06211	0.993	**0.007**	0.999			
FCUL_01092	0.226	**0.014**	0.237			
FCUL_08966	0.43	**0.035**	0.355			
Up						
FCUL_03752	0.054	0.073	0.087	0.228	**0.018**	**0.005**
FCUL_06717	**0.015**	**0.011**	**0.016**	**0.002**	**< 0.001**	**< 0.001**
FCUL_06718	**0.023**	**0.022**	**0.027**	**< 0.001**	0.598	0.3465
FCUL_06826	0.105	0.429	0.271			
FCUL_10778	**0.012**	**0.012**	**0.013**	0.292	**< 0.001**	0.079
FCUL_11936	**0.029**	**0.02**	**0.029**	0.941	**< 0.001**	0.822
Down						
FCUL_06324	**0.014**	**0.037**	**0.004**	0.444	0.098	**< 0.001**
FCUL_10992	**0.004**	**0.003**	**0.003**	0.798	**0.018**	**0.021**
FCUL_11523	**0.007**	**0.005**	**0.006**	0.368	**< 0.001**	**0.028**

Notes: [a] Comparison of gene expression between strains (S) P1P2 and UK99 subjected or not to the tebuconazole treatment (T) using two-way ANOVAs. [b] Comparison of gene expression between resistant and sensitive field strains (R) subjected or not to the tebuconazole treatment (T) using linear mixed models. *P*-values in bold indicate that the factor is significant at $P < 0.05$.
