# Supplementary material for: The adaptation of Fusarium culmorum to DMI Fungicides Is Mediated by Major Transcriptome Modifications in Response to Azole Fungicide, Including the Overexpression of a PDR Transporter (FcABC1)
Source: Front Microbiol. 2018 Jun 26;9:1385. doi: 10.3389/fmicb.2018.01385 (PMC6028722; doi:10.3389/fmicb.2018.01385)
Supplement: Table S5 — List primers used for quantification of transcript level of candidate genes. [file Table_5.PDF]

Table S5. List primers used for quantification of transcript level of candidate genes

Gene ID [a]	Description	Primer	Sequence 5' → 3'	Product (bp)
FCUL_06211	CYP51A	FCUL_06211-f	GAAGTGTCGAGAGAAGCACG	167
		FCUL_06211-r	CGCTACCAAAGACGGGGATT	
FCUL_01092	CYP51B	FCUL_01092-f	GCTCATGGAACAGAAGAAGTTCA	133
		FCUL_01092-r	AATGCCAGACTTGCCCTTGA	
FCUL_08966	CYP51C	FCUL_08966-f	TCATGGACCAAAAGAGGCTT	189
		FCUL_08966-r	GGAGGGATCCAGATGCTGTG	
FCUL_03752	Glucose transporter rco-3	FCUL_03752-f	TCACCGGATTGCTGTTCCTT	275
		FCUL_03752-r	GATGAAGTTGATACCCGTAAGC	
FCUL_06717	ABC transporter	FCUL_06717-f	TTGGTTCAGATGCAGATGCC	177
		FCUL_06717-r	GCCGACAGGGTAGTAGATGC	
FCUL_06718	Transcription factor	FCUL_06718-f	TGTTCATTTTGTGTCAAGGCAG	235
		FCUL_06718-r	TTTCTGGCGATGAACTCCCA	
FCUL_06826	Alkanesulfonate monooxygenase	FCUL_06826-f	CGAGAAGAACAACTAAGCCACG	175
		FCUL_06826-r	AGTACTTGCCCTTGTGTCCG	
FCUL_10778	Pentalenolactone D synthase	FCUL_10778-f	ATCGCGACAACGTGACTCTT	152
		FCUL_10778-r	CTCTTCAGGGAGCAACTGGG	
FCUL_11936	Ent kaurene oxidase	FCUL_11936-f	CTCCCGCCGAGGATGACTC	236
		FCUL_11936-r	TCGAGAAGACATACGCGACA	
FCUL_06324	Related to MFS1	FCUL_06324-f	CTCTTCTCCCTTGACTTGACTAT	135
		FCUL_06324-r	ACCCCATGTTGACTGGAAGG	
FCUL_10992	Short-chain dehydrogenase reductase	FCUL_10992-f	ACAGCAGCTTACGGGTATCA	131
		FCUL_10992-r	TTCCAGGCACTGTCGATACG	
FCUL_11523	Related to Rtm1p	FCUL_11523-f	CTCTTCTCCCTTGACTTGACTAT	135
		FCUL_11523-r	ACCCCATGTTGACTGGAAGG	
FGSG_06245 [b]	Cofilin	FGSG_06245-f	CCAAGAGCCGAACTGGCG	125
		FGSG_06245-r	ATCATCAGGAGACCAGGCGA	
FCUL_01337	Pre mRNA splicing factor	FCUL_01337-f	CACACCTCGTCAGAGAGCTT	146
		FCUL_01337-r	GATGATGCGTGCGTGAATGG	

Notes: [a] Gene id in *F. culmorum* (UK99) genome annotation (Urban et al. 2016).

[b] gene homolog in *F. graminearum* genome (Cuomo et al. 2007) not found in the published UK99 genome. Nevertheless, it was found in the UK99 transcriptome (this study) using a de novo assembly of the reads.
