# Supplementary material for: The adaptation of Fusarium culmorum to DMI Fungicides Is Mediated by Major Transcriptome Modifications in Response to Azole Fungicide, Including the Overexpression of a PDR Transporter (FcABC1)
Source: Front Microbiol. 2018 Jun 26;9:1385. doi: 10.3389/fmicb.2018.01385 (PMC6028722; doi:10.3389/fmicb.2018.01385)
Supplement: Table S6 — List of primers used for amplification and sequencing of FcABC1. [file Table_6.PDF]

Table S6. List of primers used for amplification and sequencing of *FcABC1*

Gene region	primer name	sequence 5' → 3'	size (bp)[a]
Promoter region			
	pro2757-f	TCGAGGTCTACCATGCTTGC	1053
	pro2757-r	TGTCGAGCTCATGTTTGCCT	
ORF and 3' UTR			
	seq2757-f	AGCCTGCAGCGGTATTTTT	5264
	seq2757i1-r	GCTTGTTCTCACGGAATGCG	
	seq2757i2-f	GACATTGCGTCTACAATCTGAGT	
	seq2757i3-r	CAACGTGGTCGAGAATGCG	
	seq2757i4-f	TGAACAGAGCTTGCTCAACAG	
	seq2757i5-r	GCTGTACGTCTTGGAGGGTC	
	seq2757i6-f	GCCGAGGGTAGTGCTAATGG	
	seq2757-r	CGCATGGCTTGGTTCACAAC	

[a] amplicon size produced by PCR using the first and the last primer for each region. Other primers were only used for sequencing
